# Artificial intelligence-guided design of high-entropy materials from bulk to nanoscale

**DOI:** 10.1039/d6na00479b

**Published:** 2026-10-10

**Authors:** Bhavana Gupta, Shashank Sundriyal, Sanjeev Kumar, Prashant Kumar

**Affiliations:** a Institute for Molecular Systems Engineering and Advanced Materials, Universität Heidelberg Heidelberg Germany; b Department of Chemistry, School of Advanced Engineering, UPES Dehradun-248007 India bhavana.gupta@ddn.upes.ac.in; c Electrical Appliances Technology Division, Central Power Research Institute Sir C. V. Raman Road, Sadashivanagar Bengaluru – 560080 Karnataka India; d Artificial Intelligence Cluster, School of Computer Sciences, UPES Dehradun-248007 India sanjeev.kumar@ddn.upes.ac.in; e Department of Physics, School of Advanced Engineering, UPES Dehradun-248007 India prashant.kumar2@ddn.upes.ac.in

## Abstract

High Entropy Materials (HEMs) have provided a breakthrough in materials science, moving beyond single-principal-element design to multielement (>5) design with multiple elements in near-equiatomic ratios. This compositional complexity introduces high configurational entropy, stabilizing single solid-solution phases and providing outstanding thermal, mechanical, and functional characteristics to alloys, oxides, carbides and nitrides. Although traditional empirical methods are the cornerstone of materials design, they are less efficient in exploring the vast compositional and processing space in the sphere of HEMs. The recent development of artificial intelligence (AI) and machine learning (ML) has altered the field of HEM research by allowing data-driven prediction of phase stability, structure–property relationships, and processing results, which has tremendously accelerated the process of materials discovery. With the combination of thermodynamic descriptors, including, but not limited to, Δ*S*_mix_, mixing enthalpy (Δ*H*_mix_), atomic size mismatch (*δ*), phase purity (*Ω*), and valence electron concentration (VEC), AI models enable researchers to perform composition and property optimization more easily than ever before. The AI-guided frameworks, despite their limitations like the lack of data, can enable autonomous and high-throughput design of HEMs. This viewpoint reflects recent developments, examples of energy and catalytic processes, and the future outlook and the potential transformative nature of AI to provide next-generation high performance entropy-stabilized materials.

## Abstract 2

High Entropy Materials (HEMs) have provided a breakthrough in materials science, moving beyond single-principal-element design to multielement (>5) design with multiple elements in near-equiatomic ratios.

## Graphical abstract

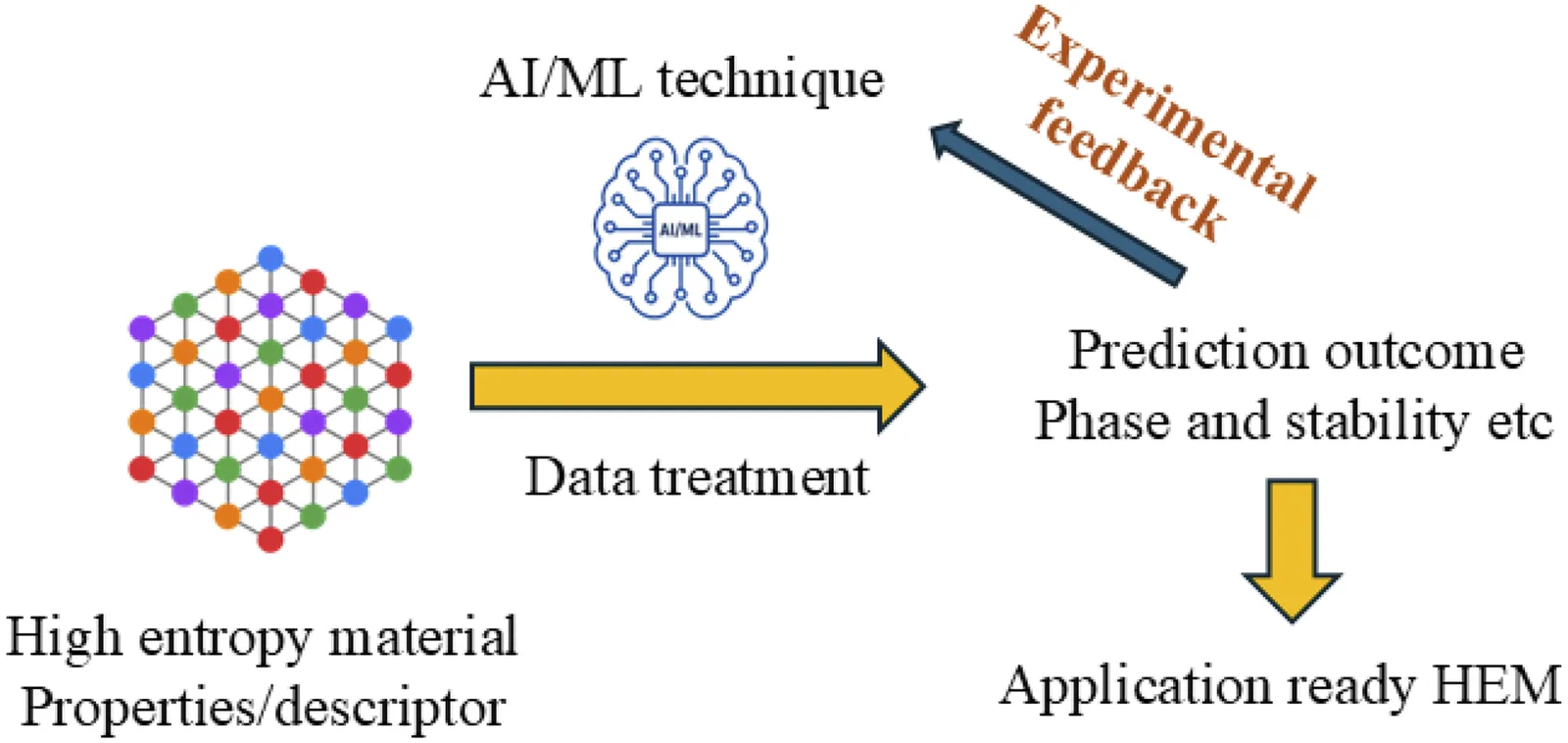

## 1 Introduction

Unlike traditional single-principal-element design strategies, high entropy materials (HEMs) are transformative.^1^ HEMs constitute a broad class of compositionally complex materials in which multiple principal elements occupy one or more crystallographic sublattices and collectively generate substantial configurational disorder. Although HEMs were initially associated with near-equiatomic compositions containing five or more principal elements, contemporary definitions also encompass non-equiatomic and sublattice-based systems in which configurational entropy, together with enthalpic interactions, atomic-size mismatch, electronic structure, local chemical ordering, and processing conditions, governs phase formation and stability, which eventually affects the symmetry.^2^ The concept was introduced in the early 2000s for high entropy alloys and since then has been extended to ceramics, oxides, carbides, borides, nitrides, and polymers. The stagnation was disrupted in 2004 by the independent, seminal studies of Cantor *et al.*^3^ and Yeh *et al.*,^4^ who proposed that concentrated alloys containing five or more principal elements in near-equimolar concentrations could stabilize into simple, single-phase solid solutions. Their unique behavior arises from four core effects: high entropy stabilization, severe lattice distortion, sluggish diffusion, and the cocktail effect, collectively leading to exceptional mechanical strength, thermal stability, and resistance to corrosion and irradiation. Owing to these properties, HEMs are promising for extreme environments and functional applications including catalysis, energy storage, and coatings.^5,6^ However, their vast compositional space necessitates advanced computational, high-throughput, and machine-learning approaches for efficient materials discovery, as shown in Fig. 1.

**Fig. 1:**
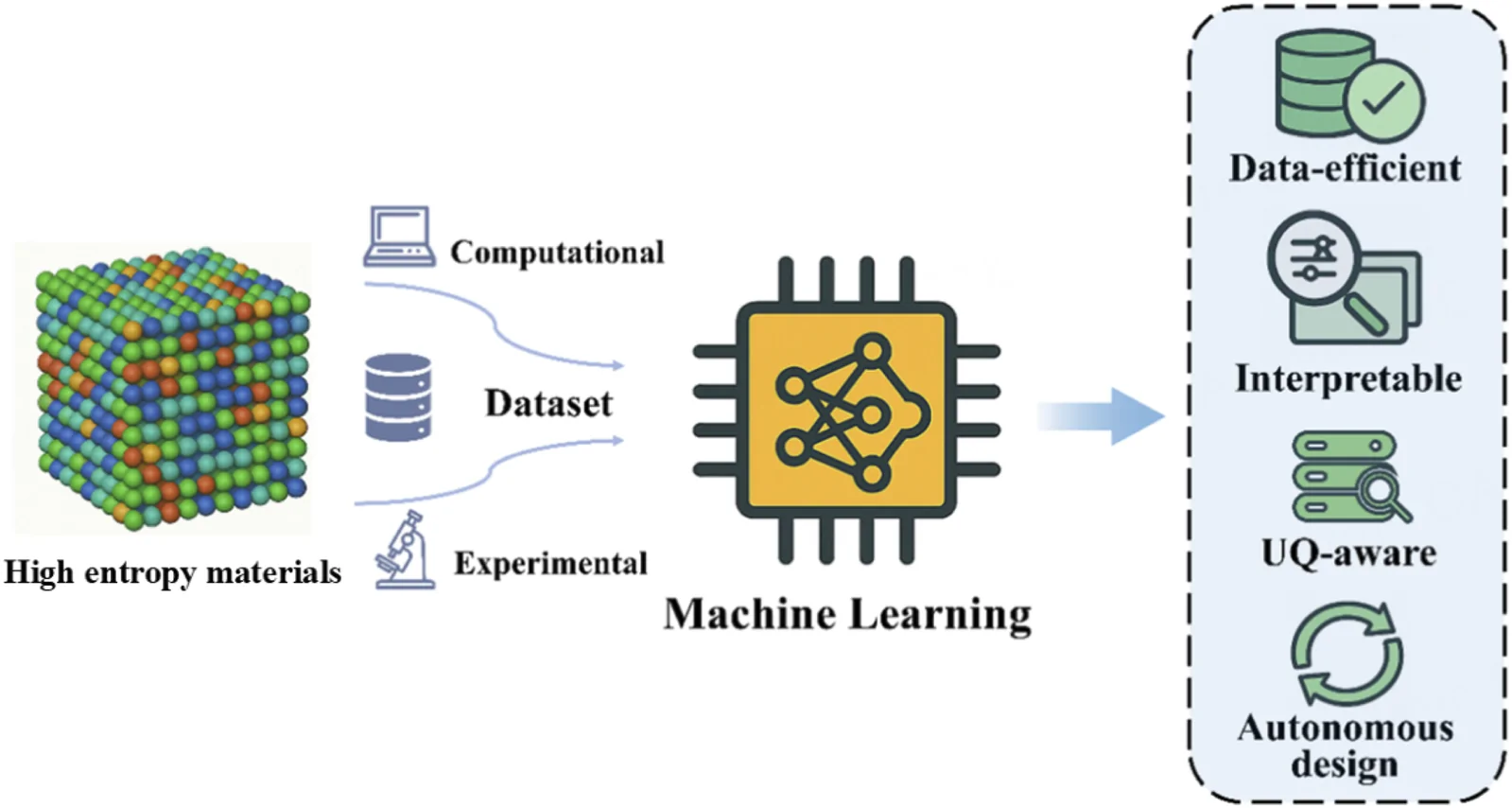
HEMs and their integration with AI/ML, highlighting various advantages. UQ, uncertainly quantification. Adapted from ref. 7 with permission from OAE Publishing Inc, copyright 2026.

Conventional HEM discovery has relied extensively on experimental trial-and-error and empirical or semi-empirical descriptors derived from composition and elemental properties.^8^ Although such approaches remain useful for initial screening, their applicability becomes limited in the exceptionally large compositional and processing space of multicomponent HEMs. Materials with similar nominal compositions may exhibit different phase constitutions and properties because of differences in elemental interactions, synthesis route, heat treatment, cooling history, microstructure, short-range ordering, and defect or interfacial states. Likewise, composition-averaged descriptors such as atomic-size mismatch (*δ*), mixing enthalpy (Δ*H*_mix_), configurational entropy (Δ*S*_mix_), the *Ω* parameter, electronegativity difference, and valence electron concentration (VEC) capture useful global trends but should not be interpreted as deterministic rules for phase stability or performance.^8,9^ These limitations, together with the relatively sparse and heterogeneous experimental coverage of the available HEM design space, motivate data-driven approaches that can learn multivariable and nonlinear composition–processing–structure–property relationships. Machine learning (ML), particularly when integrated with curated experimental data, CALPHAD, density-functional theory, and high-throughput calculations, therefore provides a complementary framework for prioritizing candidate compositions and identifying relationships that are difficult to resolve using empirical descriptors alone. Traditional descriptors derived from simpler alloy systems, including atomic-size mismatch, electronegativity difference, mixing enthalpy, configurational entropy, *Ω*, and VEC, provide useful empirical guidance but cannot fully describe phase stability across compositionally complex metallic, ceramic, and nanoscale HEMs. Their predictive value is therefore increasingly complemented by data-driven and physics-based methods capable of integrating composition, processing, structural, and local-environment information. These empirical descriptors do not usually give binary yes/no answers on the topic of phase stability in the context of 5+ element systems.^10^ HEM research has been severely changed by AI, as now researchers are able to explore composition–processing–property relationships quickly and in a data-driven manner. ML models that have been trained on experimental data, thermodynamic calculations and computational simulations can forecast phase stability and other important properties, which is of great benefit as it saves a lot of effort in experiments.^11^ High-throughput techniques, such as the calculation of phase diagrams and density functional theory (DFT), can be used to screen large compositional spaces and identify single-phase solid solutions.^8^ AI also helps to discover structure–property connections, optimize processing pathways, and control manufacturing.^12^ Although AI-based approaches face difficulties connected to data availability and model generalization, they represent a paradigm shift towards the predictive and quicker design of HEMs.

According to this view, AI is increasingly being used in the design and optimization of HEMs that are characterized by multi-principal-element compositions and large compositional spaces. It emphasizes the fact that AI/ML techniques can address the drawbacks of the traditional trial-and-error strategies by allowing the fast prediction of the phase stability, properties, and process–structure–property relationships. The article highlights the combination of AI and computational tools to fast-track the discovery of materials for different applications. It also discusses the existing challenges related to data scarcity and model interpretation and gives directions for the future development of reliable, data-driven models of next-generation high-entropy materials.

## 2 Classification of HEMs

HEMs signify the advent of a novel materials system and low-dimensional material. Conversely, traditional bulk materials base their properties on a prevailing element, and the low-dimensional materials base theirs on size and quantum confinement effects; HEMs bring about complexity through composition. HEMs produce an extremely disordered yet stable atomic environment by using several major elements in equal concentrations. This compositional freedom provides a large design space, which allows property tuning *via* elemental combinations instead of dimensional reduction and makes HEMs a separate and complementary family of highly advanced materials. HEMs are classified into various classes, each having a surface that is different based on the materials they are made of (Fig. 2a).

**Fig. 2:**
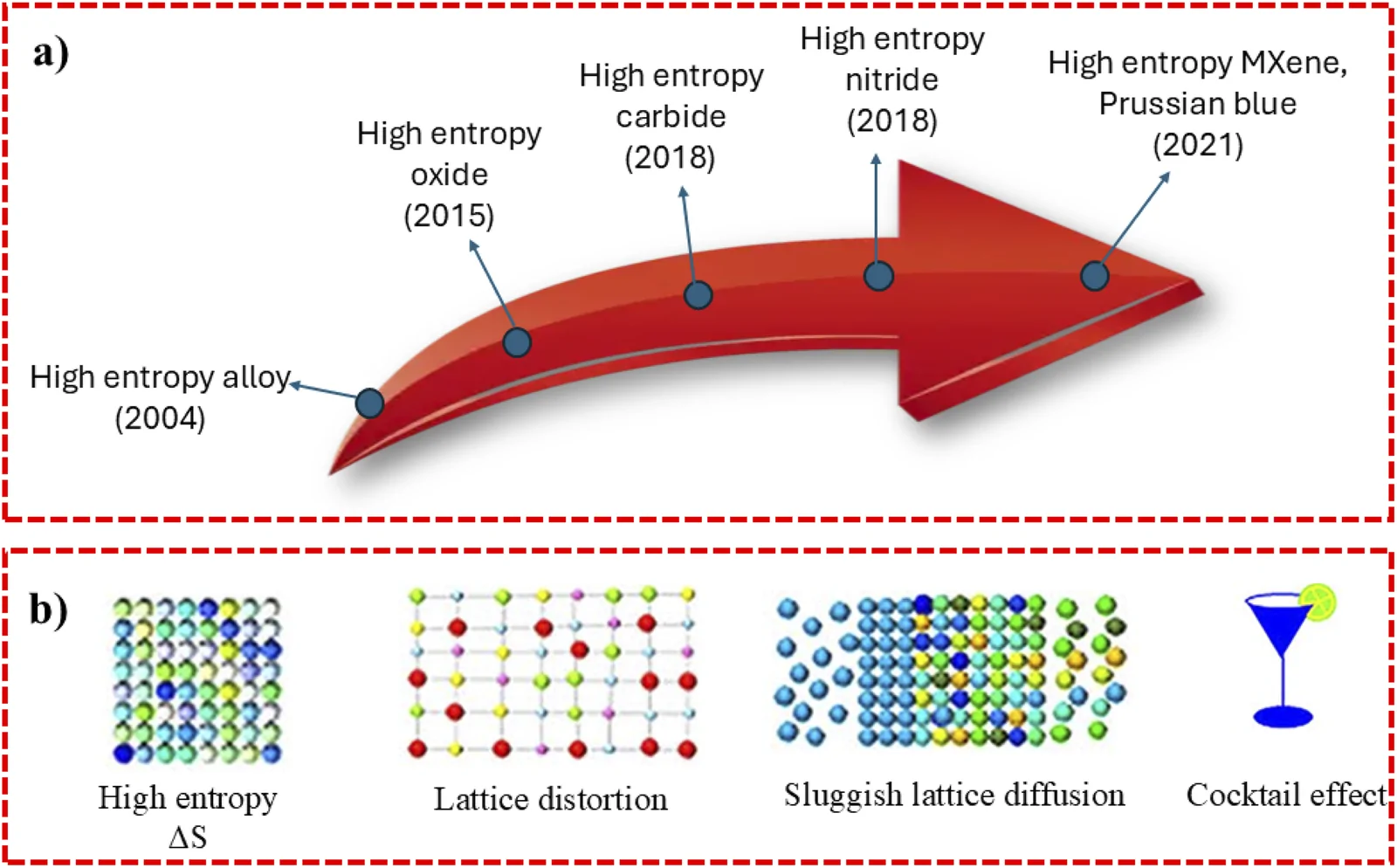
(a) Classification of HEMs with their year of origin and (b) different effects in HEMs. Adapted from ref. 13 with permission from Springer Nature, copyright 2025.

The thermodynamic and electronic descriptors of HEMs to a large extent determine the phase stability of the elements, which are combined in a multi-element manner.^14^ A key parameter is configurational entropy (Δ*S*_mix_), which quantifies the entropy brought about by numerous principal components; an increase in configurational entropy aids the formation of single-phase solid solutions by reducing the Gibbs free energy (Δ*G* = Δ*H* − *T*Δ*S*_mix_). Atomic size mismatch (*δ*) is defined as the relative difference in the atomic radii of constituent elements, which can strengthen mechanical properties by introducing lattice distortion but may disrupt the ordered structure of the intermetallic compound. The mixing enthalpy (Δ*H*_mix_) and the regular-solution interaction parameter demonstrated as *Ω* = *T*_m_Δ*S*_mix_/|Δ*H*_mix_| (where *T*_m_ is the average melting temperature), indicate the relative contributions of configurational entropy and enthalpy to the stabilization of solid solutions (Fig. 2b).

Values of *Ω* above approximately 1.1 have historically been associated with an increased tendency toward solid-solution formation in certain HEA datasets;^15^ however, this threshold should be regarded as an empirical screening guideline rather than a universal phase-stability criterion. VEC has been employed as an empirical indicator of crystal-structure preference in several transition-metal HEA families, with higher VEC values often associated with FCC stability and lower values with BCC stability. These thresholds reflect composition-dependent trends and should not be interpreted as deterministic boundaries applicable to all HEM classes.^8^ Although the above-mentioned descriptors are global (averaged across the composition), the contemporary AI designs using Graph Neural Networks (GNNs) need local features.

• Orbital-based descriptors: incorporating d-band centers and electronegativity (*χ*) on the Pauling scale to account for covalent character in high entropy carbides and oxides.

• Symmetry-adapted descriptors: in non-metallic HEMs, the features should be able to consider the ionic radii and oxidation states of the sub-lattice. According to Rost *et al.*, the charge neutrality restriction in HEOs is a constraining property that restricts the compositional space.^16^ The effect of a balance between these thermodynamic and electronic factors on phase stability in HEMs is realized. The entropic stabilization of simple solid-solution phases through a large configuration matrix, and moderate *δ*, appropriate Δ*H*_mix_, and suitable VEC determine the formation of FCC or BCC structures, which allow the design of specific properties in all mechanical, thermal, and functional applications. AI/ML is needed because conventional descriptors individually provide incomplete and composition-dependent phase information, whereas ML can integrate multiple thermodynamic, electronic, structural, and processing variables simultaneously.

### 2.1 High entropy alloys (HEAs)

They are a category of metallic materials containing multiple principal elements in nearly equal proportions, as opposed to the conventional alloy design, which is dominated by one major element, making the solid-solution phase of FCC, BCC, or HCP more stable.^17^ For AI-guided design, nominal elemental fractions are therefore useful but often insufficient descriptors. Experimentally realized properties may additionally depend on heat treatment, cooling history, grain size, phase fraction, precipitation, chemical short-range order, texture, defects, residual stress, and manufacturing route. For example, Ma *et al.* demonstrated that ageing of selectively laser-melted CoCrFeMnNi-based high-entropy-alloy composites produces nanoscale precipitates and associated microstructural changes that substantially modify the resulting mechanical response.^18^ Such observations emphasize the need to model the complete composition–processing–microstructure–property relationship rather than composition alone.

HEAs consequently represent one of the most developed HEM platforms for AI-assisted materials discovery. ML has been applied to phase classification, mechanical–property prediction, compositional screening, and active-learning-guided alloy discovery. A notable example is the closed-loop framework of Rao *et al.*, in which ML was coupled with DFT, thermodynamic calculations, and experiments to identify high-entropy Invar alloys from a very large candidate space.^11^ Thus, the principal AI challenge for HEAs is progressively shifting from simple composition–property regression toward transferable models that integrate processing history, microstructure, physical constraints, and predictive uncertainty.

### 2.2 High entropy oxides (HEOs)

They represent a new category of high-configurational-entropy-stabilized ceramic materials containing five or more metal cations in near-equiatomic ratios and forming solid solution structures such as rock-salt or spinel.^19,20^ Sivak *et al.* integrated a ML interatomic potential with physically interpretable descriptors such as mixing enthalpy and bond-length distribution to map the single-phase stability of rock-salt HEOs.^21^ More recently, thermodynamic analysis combined with ML-assisted atomistic calculations identified oxygen chemical potential and preferred cation valence as complementary descriptors controlling HEO stability and synthesizability.^22^ ML has also been used directly to screen entropy-stabilized oxide catalysts across otherwise impractically large compositional spaces.^23^ Chen *et al.* synthesized multication high-entropy tantalate and tantalate–niobate pyrochlores and showed that cation chemistry and Nb substitution influence thermal expansion, conductivity and phase stability.^24^ For AI models of high-entropy oxides and related ceramics, this motivates inclusion of sublattice occupancy, ionic size, oxidation/valence state, charge balance, oxygen-vacancy or defect chemistry and site preference rather than relying solely on metallic-alloy descriptors such as VEC. Accordingly, AI models developed for metallic HEAs should not be transferred directly to HEOs without accounting for ionic and sublattice chemistry. For oxide systems, chemically informed and structure-aware representations are particularly important for predicting phase stability, ionic/electronic transport, catalytic activity, dielectric behaviour, and other functional properties.

### 2.3 High entropy carbides/nitrides (HECs/HENs)

These are a new subclass of HEMs, consisting of five or more metal cations in near-equiatomic ratios with carbon or nitrogen anions, and use high configurational entropy to stabilize crystal structures with simplicity and phase homogeneity,^25,26^ in spite of the chemical complexity. Experimental and first-principles studies have shown that their phase formation and properties depend on compositional disorder, lattice distortion, electronic structure, elemental miscibility, processing temperature, and sublattice chemistry.^26,27^ Harrington *et al.*, for example, combined an *ab initio* entropy-forming descriptor with experimental synthesis to screen phase stability in a series of five-metal high-entropy carbides and demonstrated relationships between phase formation, electronic structure, and mechanical properties.^27^ Bulk HENs and HECs have likewise been shown to exhibit strongly composition- and processing-dependent single- and multiphase formation.^26^

These characteristics make HECs and HENs particularly suitable for physics-informed and data-driven screening. Their AI representations may include elemental fractions together with thermodynamic descriptors, lattice mismatch, valence-electron characteristics, local bonding information, carbon/nitrogen stoichiometry, vacancy or defect information, and physics-based quantities obtained from DFT or atomistic simulations. Mitra *et al.* demonstrated an ML framework for predicting single-phase formation in high-entropy carbides using thermodynamic and structural descriptors while explicitly addressing the limited and imbalanced nature of available ceramic datasets.^28^ More recently, Zhang *et al.* developed an experimentally coupled ML framework for high-entropy carbide phase prediction that combined literature data, feature selection, data-imbalance treatment, uncertainty-guided candidate selection, and experimental feedback.^29^

These studies demonstrate that AI can accelerate high-entropy ceramic discovery, but they also highlight a key limitation: available datasets remain substantially smaller and less standardized than those for many conventional materials. Reliable models therefore require careful treatment of data imbalance, uncertainty, processing metadata, chemical applicability domain, and experimental validation.

## 3 High entropy nanomaterials

Particle size and shape, together with phase and content, are important factors in influencing the catalytic performance of HEMs when they are produced at the nanoscale. These factors have a significant impact on the material's electronic structure and surface characteristics, which in turn control its catalytic action (Fig. 3a and b). Particle size and shape, in particular, can alter the electronic states and surface binding energies, which ultimately impact the adsorption and activation of reactant molecules. The Sabatier principle states that an effective catalyst should have an ideal binding strength that promotes both reactant activation and product desorption, rather than interacting with the reactants too strongly or too weakly. As a result, the connection between catalytic activity and adsorption energy is frequently volcano-like, with the highest catalytic performance occurring at an intermediate adsorption strength. In contrast, HEMs provide various catalytic activity scenarios due to the various combinations of elements that are present on the surface.^30^ The HER, in which the reaction intermediate, H*, binds to a surface with an inhomogeneous composition, is a common example. Because the local electronic environment varies and certain sites encourage adsorption while others aid in desorption, the resulting catalytic activity has a Gaussian distribution.^31^

**Fig. 3:**
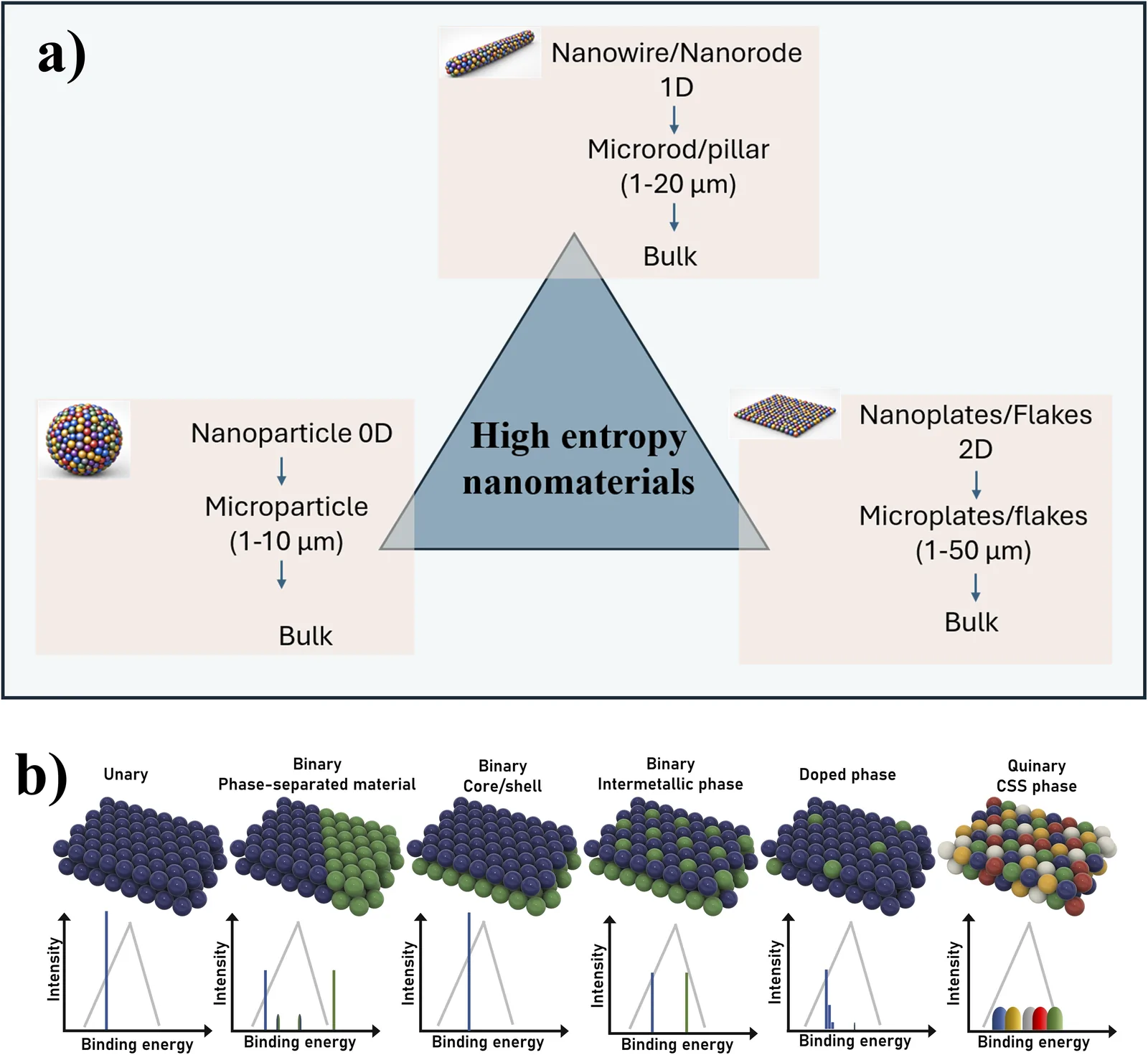
(a) HEMs of various dimensionalities image created with AI; (b) different types of catalyst surfaces with corresponding patterns of binding energies. Reproduced from ref. 32 with permission from John Wiley and Sons, copyright 2021.

Because this intermediate is easily transferred from one site to another, the diffusion barrier of H* is reduced as much as possible. Overall, composition is one of the most effective ways to modify the system's electronic structure and d-orbital energy. Furthermore, the reactant's surface binding energy is ultimately affected by the elements' interaction, which can be either long-range or short-range.

Researchers can typically create the adsorption energy relationship by choosing several materials with varied compositions, but the large compositional space makes optimization challenging. The need for noble metals can be reduced by using a variety of metals. For example, CrMnFeCoNi is used more frequently for the oxygen reduction reaction than the well-known Pt-based catalyst.^33^ CoMoFeNiCu was also found to be helpful in the decomposition of ammonia.^34^ These studies demonstrate the potential for compositional engineering to produce catalysts free of noble metals that are more efficient and less expensive. Particle shape plays a crucial role in customizing the catalytic properties in addition to composition. While the hollow void at the nanoscale dimension increases the surface area for catalytic reactions, the porosity of small dimensions aids in the creation of the channel. Through the application of various porous supports for their dispersion, the adaptability of HEMs with increased catalytic activity and surface area evolved. As a result, creating a porous framework was less difficult.

Lastly, another benefit of creating nano dimensions is the particle form, which might offer a particular crystal aspect. The catalytic site varies because different facets have distinct atomic configurations and surface energies. Strong catalytic sites are typically created by the formation of high index facets with low coordinated atoms. Varied crystal facets are produced by the HEM nanoparticles of various materials using the same synthesis technique, which ultimately leads to varied catalytic activity. For example, convex-cube PtFeNiCuMoRu nanoparticles and Pd@PtRuFeCoNi nanocubes have been manufactured with varied crystal facets, but controlling the high index crystal facets of HEMs is still difficult because of their high surface energy; thus, this has become the focus of future studies.^35^ In general, four distinct factors composition, particle size, shape, and facets affect catalytic activity in nanostructured HEMs. Working on the intermediate reaction and reaction mechanism is therefore essential. Even though there has been considerable progress in this area, it is still difficult to produce highly effective catalysts on a large scale at a reasonable cost.

## 4 AI/ML in HEM research

AI-driven HEM research requires an integrated ecosystem linking materials data, representation, ML and physics-based validation. General first-principles repositories such as the Materials Project, OQMD and AFLOW provide computed structural, thermodynamic, electronic and related property data for descriptor construction, screening and model development.^36–38^ COD’HEM adds a dedicated HEM-specific source compiled from the published literature.^39^ At the software level, pymatgen supports structure/composition objects, phase-diagram and electronic-structure analysis, file conversion and database interoperability, while matminer converts composition, structure, site and electronic information into numerical descriptors and supplies benchmark-ready materials datasets.^40^ ASE provides an interoperable Python layer for constructing, executing and analysing atomistic simulations.^41^ All this information is summarized in Table 1.

**Table 1:** Data resources and software ecosystem for AI-driven high-entropy materials

Resource	Type	Main information/use	Relevance to HEM-AI
Materials Project^36^	Database	Computed structures/properties; REST API for thermo, electronic, elastic, surface and related data	DFT descriptors, stability screening, transfer/pretraining and comparison with HEM calculations; retain calculation provenance
OQMD^37^	Database	Large DFT thermodynamic/structural database; composition search, phase diagrams, REST and OPTIMADE	Formation energies, convex-hull analysis and automated chemical-space screening
AFLOW^38^	Database/workflow	High-throughput structural, energetic, electronic and elastic data; REST API/AFLUX	Prototype search, stability screening and automated feature/data retrieval across broad chemistry
COD’HEM^39^	HEM-specific database	Literature-curated HEA/HEM composition, phase and experimental property information	Direct HEM benchmark/property source complementing general DFT repositories
Pymatgen^42^	Materials library	Structures/compositions, phase diagrams, symmetry, electronic analysis, file I/O and MP integration	Consistent HEM structure representation and physics-based descriptor generation
Matminer^40^	Featurization/datasets	Materials datasets and featurizers for composition, sites, structures, band structure and DOS	Converts HEM chemistry/structure into ML-ready descriptors and supports benchmarking
ASE^41^	Atomistic workflow	Build, manipulate, run and analyse atomistic simulations across many calculators	Connects DFT, MD and ML potentials for HEM data generation and validation

Because these resources differ in provenance and methodology, values should not be pooled uncritically. Reproducible HEM-AI studies should report database version and access data, software version, filtering criteria and preprocessing, and where computed data are used the relevant calculation settings. Experimental records should retain composition, processing, microstructure, test conditions and uncertainty wherever available.

By treating the composition and processing parameters as a “genome,” we can now employ AI to decode the complex relationships that govern the next generation of superalloys and functional ceramics, effectively bypassing the limitations of human-centric observation and traditional thermodynamic modeling. The relationship between properties and composition in optimization requires a lot of resources and time, which can be overcome by AI/ML techniques. ML techniques have potential to analyze a large number of datasets to understand the possible relationships between properties, processing, structure and composition which are generally difficult to detect by simple conventional methods.^43^ There is immense potential for AI to be implemented for structural design optimization of HEMs. Fig. 4 depicts AI-driven structural design of HEMs.

**Fig. 4:**
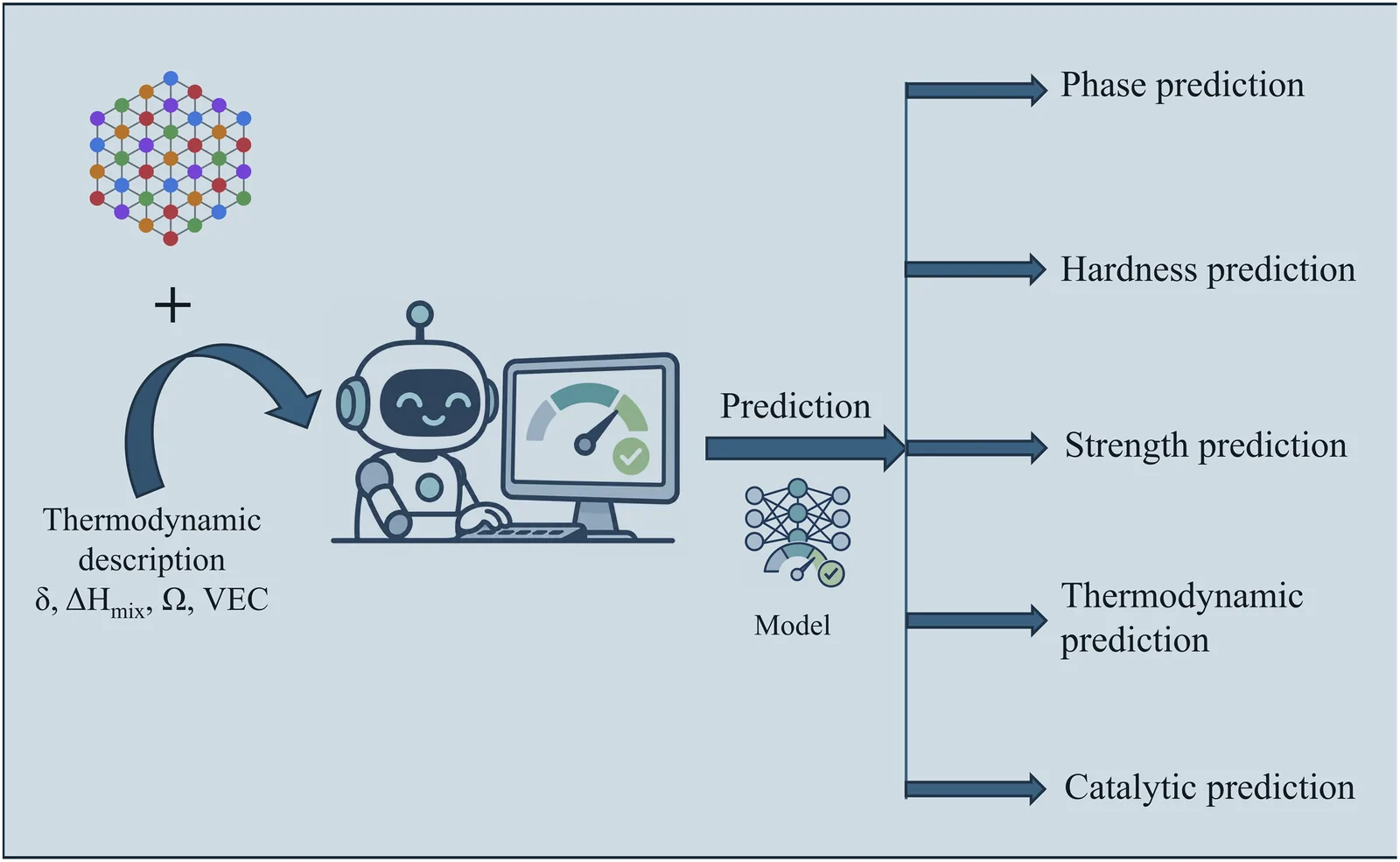
AI-driven structural design of HEMs image created with AI.

Recent research has clearly demonstrated that AI and ML techniques are being used to accelerate the discovery of HEMs by predicting key properties, such as phase stability and mechanical performance, at significantly lower costs compared to traditional experimental approaches and first-principles theoretical methods.^44^ Globally, AI and ML techniques facilitate the identification of material properties, uncover hidden patterns in data, optimize composition and synthesis processes, and integrate with high-throughput experimental methods.^45^ The following techniques are known for HEM property estimation and prediction.

### 4.1 Supervised learning

In this approach, computers learn from labeled data, meaning that input data are provided to a model along with the corresponding correct outputs. Based on the mapping between inputs and outputs, models are trained with help of mathematical functions, enabling them to predict accurate results for previously unseen data. The process begins by generating labeled data, which are then divided into two categories *i.e.* training and test sets. Of these two sets, the model is trained on the training set, and its predictions are compared with the labeled outputs to calculate an error, as shown in Fig. 5a.

**Fig. 5:**
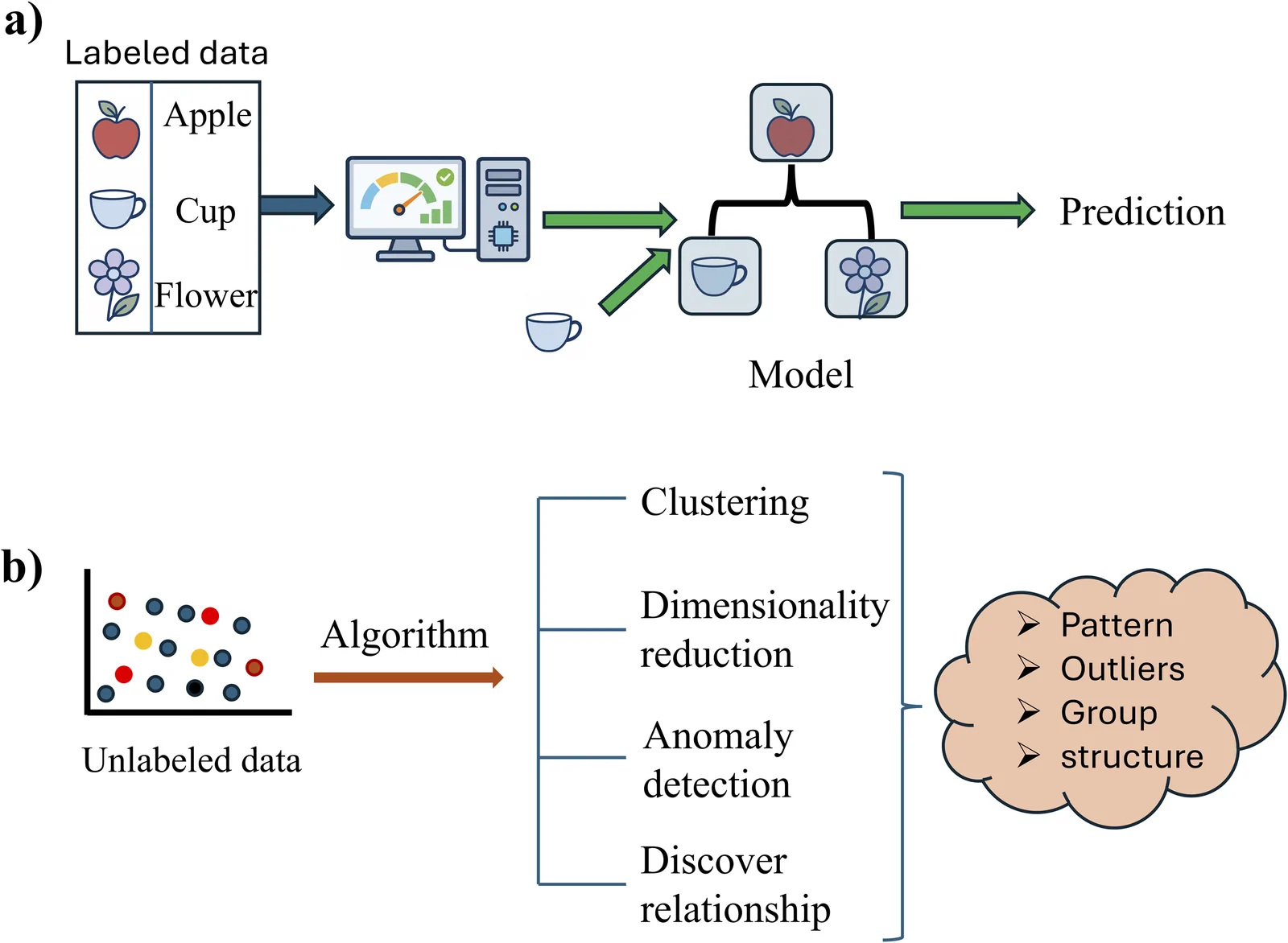
AI/ML model creation by (a) supervised and (b) unsupervised learning image created with AI.

Model parameters are iteratively adjusted to minimize this error, and each complete repetition of this training cycle is referred to as an epoch. Once trained, the model is evaluated on new data for either classification or regression tasks. Classification involves the prediction of categorical output (*e.g.* yes/no decisions), whilst regression is the prediction of continuous values, *e.g.* temperature within a given range. In the computational design of HEAs, a major problem is the phase stability classification that is particularly to separate between single-phase solid solutions (SS), multi-phase mixtures, and amorphous metallic glasses. In supervised learning, there are two large paradigms that are typically used. Other widely used algorithms in classification tasks are logistic regression, decision trees, support vector machines (SVMs), and artificial neural networks (ANNs). Linear regression, random forest regression, gradient boosting, and neural networks are commonly used in the case of regression tasks. The phase formation prediction, as well as mechanical property estimation and thermal stability analysis in HEAs, has been widely employed with these supervised learning techniques. One of them, eXtreme Gradient Boosting (XGBoost), has become a popular technique to predict the HEA phase, especially in cases when dealing with structured tabular data. It is effective because it is able to make sense of the complex data with non-linear relationships and feature interactions without necessarily requiring very large datasets.^46^ It is also applied in predicting the mechanical properties with great accuracy.^47^ Graph Neural Networks (GNNs) are a transition to the concept of representation learning. The nodes (*V*) of an HEA in this paradigm are the atoms, and the edges (*A*) are the chemical bonds or spatial proximities in this paradigm. The primary trade-off is that GNNs require significantly more data and precise structural information *e.g.*, atomic coordinates from DFT compared to XGBoost. Comparison of both the techniques is shown in Table 2. Physics-based simulation can be used to generate structured training data in HEM domains where comprehensive experiments are difficult to obtain. For example, Nie *et al.* combined molecular-dynamics nanoscratch simulations with supervised ML for non-equiatomic FeCoCrNiMn HEAs, using composition-dependent simulation outputs to predict friction and wear behavior and SHAP analysis to identify influential elemental contributions.^48^ Such hybrid workflows illustrate how atomistic calculations can supplement sparse experimental observations, although model validity remains bounded by the fidelity and chemical coverage of the underlying simulation data.

**Table 2:** Comparative functionality of XGBoost and GNN AI/ML techniques

Feature	XGBoost/random forest	GNN
Data requirement	Low to moderate (∼100 s of samples)	High (∼1000 s of samples or DFT)
Input type	Manual (*δ*, VEC, Δ*H*_mix_) descriptors	Raw crystal structure/graph
Interpretability	High (feature importance/SHAP)	Low (“black box” latent space)
Sensitivity	Global compositional trends	Local atomic environment and SRO
Best for	Rapid screening experimental data	High-fidelity property prediction (DFT-led)

### 4.2 Unsupervised learning

In unsupervised learning there is no predetermined output label or category. To illustrate this, we can take a set of 1000 customers: the age, income, and frequency of purchase are known, but the type of customer is not known. Unsupervised learning techniques are employed in these situations to discover submerged patterns in the data (Fig. 5b). The general process of the work includes missing data cleaning, data normalization, and working with categorical data by changing them to numerical form. Typical unsupervised learning algorithms include clustering algorithms (*e.g.*, K-means and hierarchical clustering), dimensionality-reduced algorithms (*e.g.*, principal component analysis, PCA) and anomaly detection algorithms. These algorithms can find patterns by clustering similar data points, reducing dimensionality to reveal underlying structures, or detecting unusual observations. Patterns can be extracted and analyzed to give meaningful results. For example, the customers can be divided into various groups, including budget shoppers, luxury buyers, and occasional buyers. Within the framework of HEMs, unsupervised learning is used to determine composition–property correlations, group alike alloy systems, and determine phase formation patterns. It has been a common method to determine and study the major driving factors of phase formation in multicomponent alloys.^49^

### 4.3 Deep learning

Deep learning is one of the advanced ML techniques that employ multilayer neural networks to model non-linear and complex relationships between input and output parameters. It is widely used for image analysis, natural language processing, and speech recognition. The process begins with data collection, followed by noise removal, normalization, and conversion of data such as images into numerical form. The prepared dataset is then divided into training, validation, and test sets, as shown in Fig. 6a. Different deep learning architectures serve different purposes. For example, Convolutional Neural Networks (CNNs) are generally used for the analysis of images, Recurrent Neural Networks (RNNs) and Long Short-Term Memory (LSTM) networks are suited for sequential or time-series data, and transformer models are designed for language processing and large-scale AI applications. Within the network, input data pass through multiple layers, where features are progressively extracted, ultimately producing a prediction. This prediction is compared with the true or expected output to compute the error. The error is then minimized through backpropagation, during which each layer updates its weight using optimization algorithms such as Gradient Descent or the Adam optimizer. Such deep learning approaches have been applied to model atomic interactions and predict the thermodynamic and mechanical properties of HEMs.^50^

**Fig. 6:**
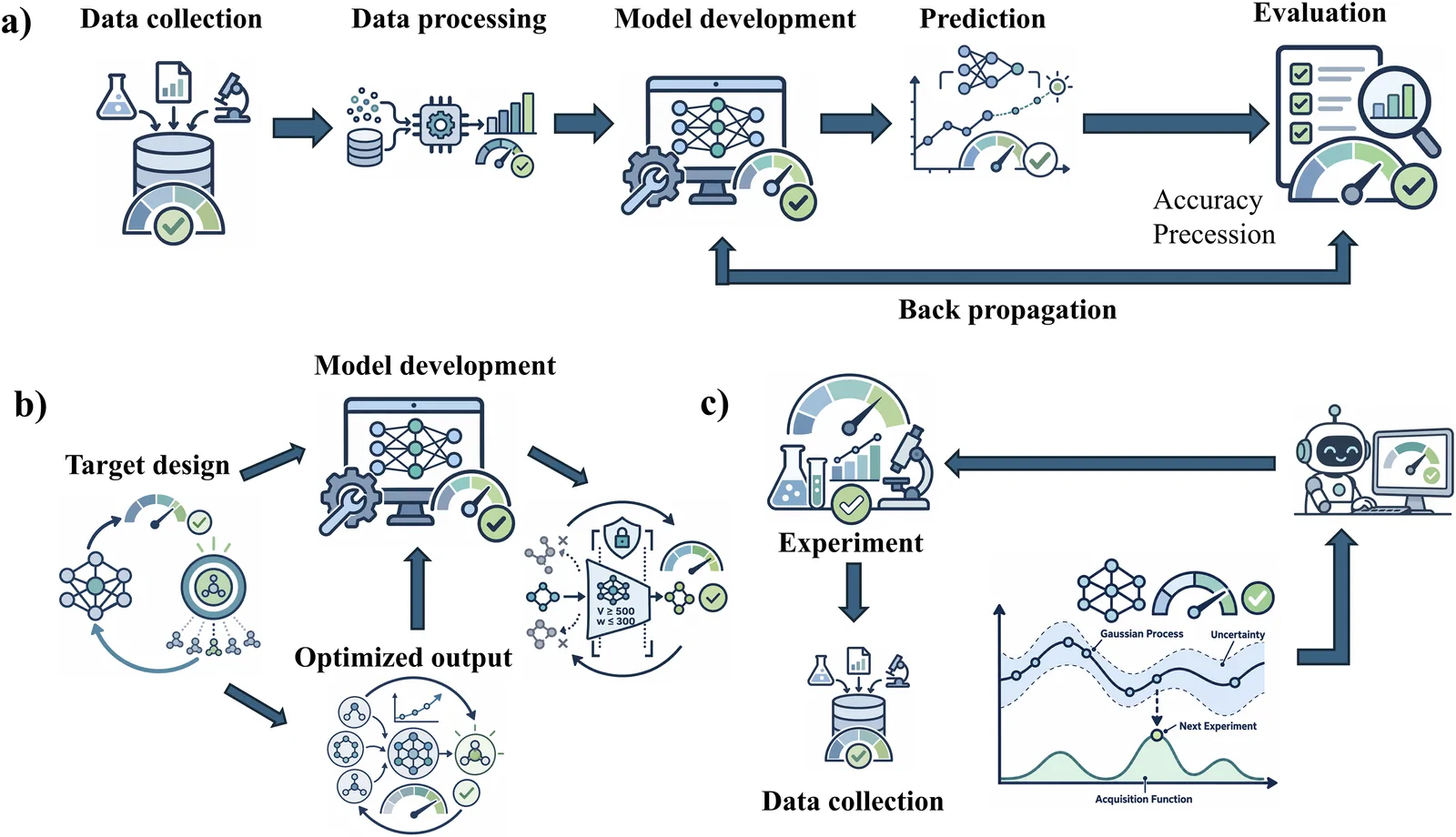
AL/ML models based on (a) deep learning, (b) generative model inverse design, and (c) active learning and Bayesian optimization image created with AI.

### 4.4 Generative models and inverse design

While supervised learning primarily models the forward relationship between composition and properties, the vast “compositional wilderness” of HEMs calls for a reverse strategy. Inverse design employs generative artificial intelligence (AI) to explore the N-dimensional compositional space by beginning with a desired property profile, such as a target yield strength or catalytic activity, and then generating chemical compositions and structural configurations that satisfy those criteria (Fig. 6b). Generative models learn the underlying probability of distribution of existing data in order to create new, similar samples. Rather than performing simple classification or regression, these models can generate novel data in the form of images, molecules, material structures, or even text. The process typically involves encoding existing data into a latent space representation, where meaningful patterns, structures, and relationships are captured. This latent space is then systematically adjusted or optimized until the generated candidates meet the desired target properties. The generated structures can subsequently be evaluated through theoretical simulations and experimental validation. Feedback from these evaluations is used to iteratively update and refine the model, improving its predictive accuracy and accelerating the discovery process. The combination of targeting the desired properties and backpropagation to identify suitable compositions or structures has made inverse design a powerful paradigm in materials science, drug discovery, photonics, and catalysis. It represents one of the fastest emerging approaches for materials and molecular discovery, with the added advantage of uncovering unexpected and innovative solutions. Different types of models such as variational autoencoders (VAEs), generative adversarial networks (GANs) and diffusion models and transformer models are approaches used in generative models and inverse design.

A VAE consists of an encoder that maps an input material representation, *x*, such as compositional or structural descriptors, to an approximate latent-variable posterior *q*_*φ*_(*z*|*x*), and a decoder that reconstructs or generates material representations through *p*_*θ*_(*x*|*z*). Unlike a conventional autoencoder, the latent representation in a VAE is probabilistic, enabling continuous sampling and interpolation within the learned design space.^51^

The VAE is trained by maximizing the evidence lower bound (ELBO) on the log-likelihood of the observed data:^51^1
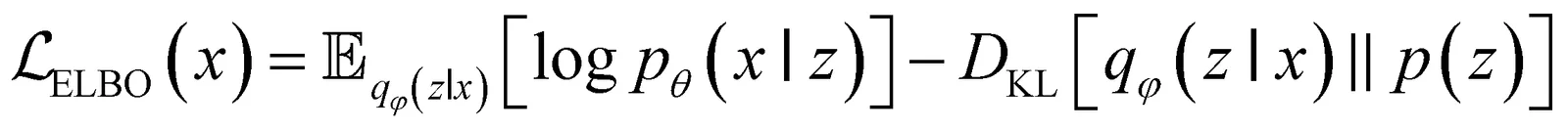
where *q*_*φ*_(*z*|*x*) is the approximate posterior distribution generated by the encoder, *p*_*θ*_(*x*|*z*) is the decoder likelihood, and *p*(*z*) is typically chosen as a standard normal prior. The first term is the expected reconstruction log-likelihood and encourages accurate reconstruction of the input representation. The second term is the Kullback–Leibler (KL) divergence, which regularizes the approximate posterior toward the prior and promotes a smooth, structured latent space.^51^

For inverse design of HEMs, a trained generative model can map known compositions and property descriptors into a continuous latent representation and subsequently search for representations that satisfy desired property targets. Generative deep-learning approaches have already been explored for inverse design of high-entropy refractory alloys, illustrating the potential of latent-space exploration for composition discovery.^52^ However, generated candidates should subsequently be screened for physical constraints, thermodynamic stability, synthesizability, applicability domain, and ultimately computational or experimental validation. Inverse design should optimize feasible materials rather than unconstrained numerical objectives. Generic constrained-optimization methods, such as the best-of-L local-search/contractive-consensus framework of Mohammadi *et al.*, illustrate how explicit bounds and constraints can be embedded into an iterative search.^53^ In HEM applications, however, the constraints must be materials-specific and may include elemental-fraction normalization, phase stability, charge neutrality, processing windows, elemental criticality, cost and synthesizability. Similarly, the CNT-focused AI review is useful only as a nanomaterials analogue for the principle that inverse-designed candidates require feasibility screening, synthesis-aware modelling, characterization and iterative experimental feedback.^53^ GANs utilize a game-theoretic framework to produce novel HEMs. In this architecture, a Generator (G) synthesizes new material candidates, while a Discriminator (D) evaluates them by attempting to differentiate generated samples from “real” structures validated through DFT calculations or experimental data. In HEM research, GANs have been applied to learn compositional patterns from existing datasets and subsequently propose new candidate compositions with promising properties.^50^ For inverse design, this method has also been used to create alloys, which are further validated by theoretical simulations and experiments.^52^ A comparison between the GAN and VAE techniques is illustrated in Table 3.

**Table 3:** Comparative functionality of GAN and VAE AI/ML techniques

Technique	Generative process	Strengths	Weaknesses
VAE	Latent space interpolation	Smooth, continuous manifold; good for optimization	Can produce “blurry” or non-physical structures
GAN	Adversarial competition	Produces high-fidelity, discrete crystal structures	Prone to “mode collapse” and training instability

As the field matures, the integration of Physics-Informed Neural Networks (PINNs) into generative frameworks ensures that AI-designed HEMs adhere to thermodynamic constraints, such as the convex hull of stability, effectively bridging the gap between “black box” generation and metallurgical reality.^54^

### 4.5 Active learning and Bayesian optimization

The discovery of HEMs is seldom driven by a single objective. In practice, engineering applications require the simultaneous optimization of competing properties, most prominently the strength–ductility trade-off. Within the vast, high-dimensional compositional space, identifying the Pareto front the set of optimal solutions where improving one property necessarily compromises another demands an advanced iterative strategy known as active learning (AL) (Fig. 6c). The enormous compositional and processing space of HEMs makes exhaustive experimental or first-principles exploration impractical. Active learning (AL) and Bayesian optimization (BO) are complementary sequential-learning strategies that can reduce the number of expensive experiments or simulations required during materials discovery. Although both use information acquired from previous evaluations to determine subsequent samples, their primary objectives are different, and they should not be treated as synonymous methodologies.^55,56^

#### 4.5.1 Active learning

Active learning is primarily concerned with improving the predictive model by selecting the most informative new samples for labelling, simulation, or experimental evaluation. In materials science, uncertainty-aware adaptive sampling has been widely discussed as a route to efficiently expand sparse datasets and target informative regions of the design space.^55^ Starting from an initial labelled dataset2
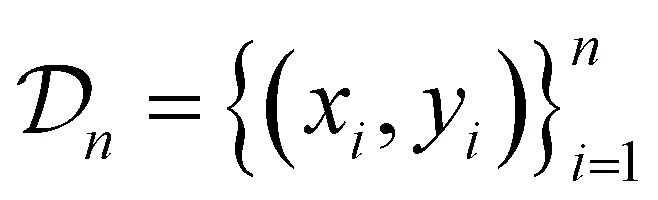
a predictive model is trained and used to identify candidate *x* values expected to provide the greatest information about the underlying composition–processing–structure–property relationship. Candidate selection may be based on predictive uncertainty, disagreement among ensemble models, expected model change, diversity, or related informativeness criteria.3
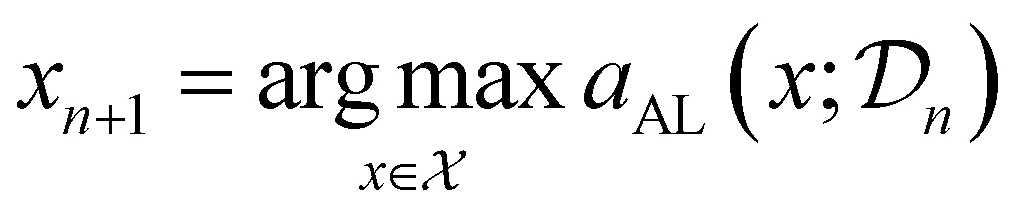
where the active-learning query function measures the informativeness of candidate *x*. The selected candidate is then evaluated experimentally or computationally, the resulting label is added to the dataset, and the model is retrained:4
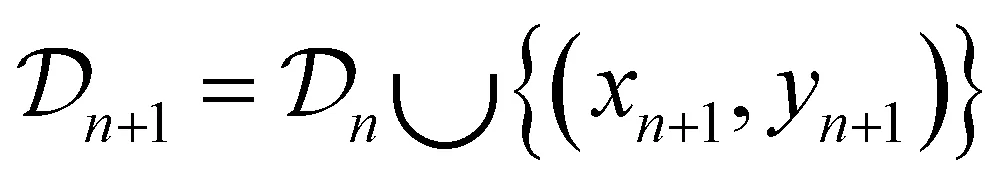


This approach is particularly valuable for HEMs because experimental characterization and high-fidelity calculations are expensive, while the labelled chemical space remains sparse. A notable example is the ML-enabled discovery of high-entropy Invar alloys, where an active-learning closed loop integrated ML with DFT, thermodynamic calculations, and experiments to navigate a very large compositional space.^11^

#### 4.5.2 Bayesian optimization

Bayesian optimization, in contrast, is primarily an objective-directed search strategy. Its purpose is not necessarily to maximize global predictive accuracy, but to identify candidate conditions or compositions that optimize an expensive objective function using as few evaluations as possible. BO constructs a probabilistic surrogate for the unknown objective and uses an acquisition function to select the next evaluation point by balancing exploration and exploitation.^56,57^

Gaussian process models are frequently used as surrogates because their posterior predictive distribution provides both an expected value and an uncertainty estimate. At iteration *n*, the next candidate can be expressed generically as:^56^5
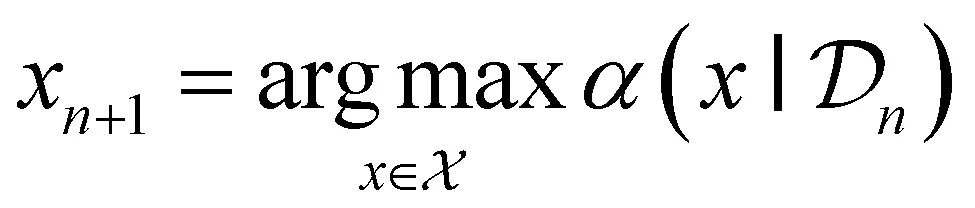
where *α* denotes the acquisition function conditioned on the current observations. Common acquisition functions include Expected Improvement (EI), Probability of Improvement (PI), Upper Confidence Bound (UCB), and entropy- or information-based criteria.^56,57^ For example, a UCB-type acquisition function for a maximization problem can be written as:6
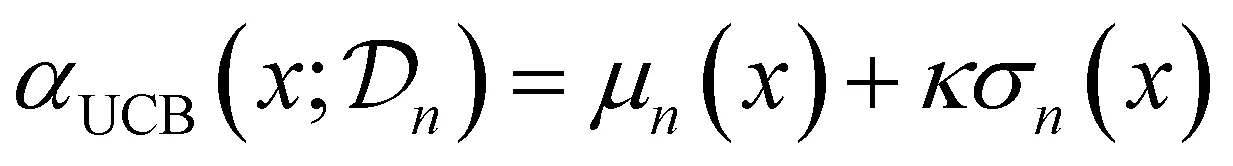
where *κ* > 0 controls the relative emphasis placed on exploration of uncertain regions *versus* exploitation of regions with high predicted performance. The classical expected-improvement framework is especially relevant when objective evaluations are costly and only a limited number of experiments or simulations can be performed.^57^

#### 4.5.3 Multi-objective Bayesian optimization

HEM design is frequently multi-objective because improvements in one property may compromise another, as occurs in strength–ductility, activity–stability, density–strength, or performance–cost trade-offs. Multi-objective Bayesian optimization (MOBO) extends BO to vector-valued objectives:7
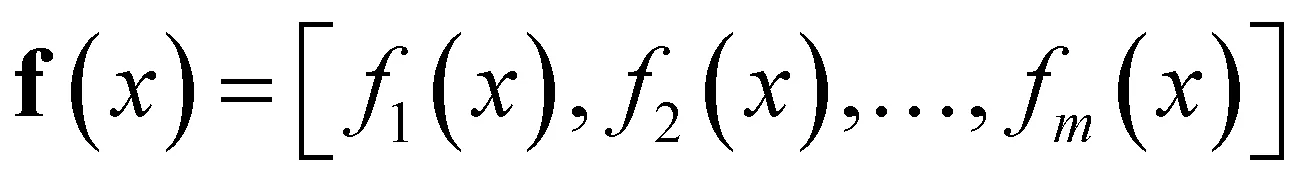
with the aim of efficiently identifying a Pareto-optimal set in which no objective can be improved without degrading at least one other objective. Hypervolume-based acquisition strategies, including expected hypervolume improvement, are widely used to prioritize candidates expected to expand the currently known Pareto front.^58^ This framework is particularly appropriate for HEM discovery when multiple functional, mechanical, economic, and synthesizability constraints must be considered simultaneously. In HEAs, this has led to the discovery of “transformation-induced plasticity” (TRIP) and “twinning induced plasticity” (TWIP) alloys that bypass the traditional Banovic curves of metallurgical failure.^59^ Closed-loop discovery relies on a paradigm in which the AI (the “brain”) directs robotic synthesis and characterization platforms (the “hands”) without human intervention. Active learning can drastically reduce the number of candidates that require high-cost experimental evaluation, although the reduction is problem-dependent and should not be expressed as a universal percentage. In a direct HEA demonstration, Rao *et al.* ML, DFT, thermodynamic calculations, and experiments in a closed loop; only 17 new alloys were processed and characterized from a compositional space containing millions of possible candidates, leading to the identification of two high-entropy Invar alloys with very low thermal expansion.^11^ AL preferentially selects samples that are expected to improve the model, whereas BO preferentially selects samples that are expected to improve the objective. In a practical HEM workflow, the two may be combined. For example, active learning may first reduce uncertainty in poorly sampled chemical regions, after which BO targets compositions that satisfy specific performance objectives.^55,56^

### 4.6 Reinforcement learning (RL)

RL provides a sequential decision-making framework in which an agent learns how to select actions through repeated interaction with an environment. Unlike conventional supervised learning, which primarily learns a mapping from a fixed set of inputs to outputs, RL optimizes a sequence of decisions by considering the cumulative consequences of these decisions. This feature is potentially valuable for HEMs, where discovery and processing often involve sequential changes in composition, synthesis conditions, microstructure, or atomic configuration across a very large design space.^60,61^

#### 4.6.1 HEM discovery as a Markov decision process

An RL problem is commonly formulated as a Markov decision process (MDP):8
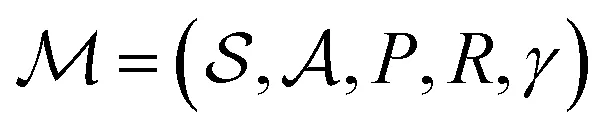
where 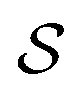 is the state space, 
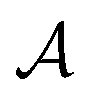
 is the action space, *P* describes the state-transition dynamics, *R* is the reward function, and *γ* is the discount factor, with 0 ≤ *γ* ≤ 1.^60^ For HEM discovery, these quantities should be defined using physically meaningful materials variables rather than generic RL examples.


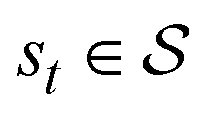
the state represents the information available to the agent at decision step *t*. Depending on the application, it may contain the current elemental composition; phase constitution; thermodynamic or electronic descriptors such as Δ*S*_mix_, Δ*H*_mix_, atomic-size mismatch *δ*, *Ω*, and VEC; processing history; microstructural descriptors such as grain size, phase fraction, precipitation state, porosity, or residual stress; measured or predicted properties; and model uncertainty. For high-entropy nanomaterials, the state may additionally include particle size and morphology, local atomic environments, surface composition, segregation, oxidation state, or atomic coordinates.^62,63^ The exact state definition should be application-specific; no single state vector is universal for all HEM problems.


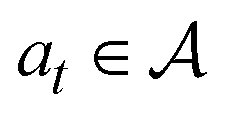
: an action represents a physically feasible modification of the current material or process. Examples include changing an elemental fraction, substituting a constituent element, modifying annealing temperature or dwell time, adjusting cooling rate, changing deposition or additive-manufacturing conditions, or rearranging atoms in a multicomponent nanocluster. For compositional design, the action space should preserve basic constraints such as:9
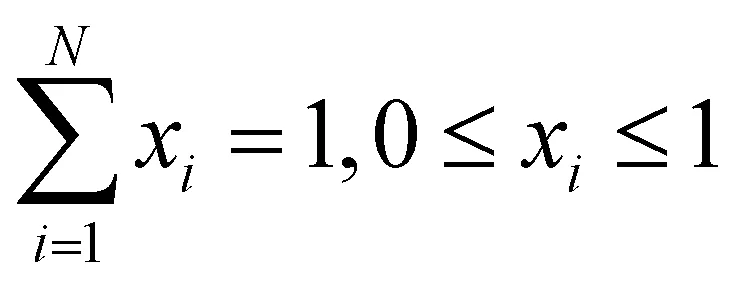


Additional constraints may include charge neutrality, accessible processing windows, elemental compatibility, cost, toxicity, critical-element content, and synthesizability.

After action a_*t*_ is applied in state s_*t*_, the environment produces a new state according to

10 s_*t*+1_ ∼ *P*( ·∣s_*t*_,a_*t*_ )

and the agent receives a reward

11 *r*_*t*+1_ = *R*(s_*t*_,a_*t*_,s_*t*+1_)

In an HEM workflow, the environment may be a physical experiment, CALPHAD calculation, DFT, molecular dynamics (MD), a validated machine-learning surrogate, or an automated synthesis–characterization platform. Model-based RL can therefore learn through a computational or surrogate environment before, or together with, higher-cost experimental evaluation.^61^

The reward translates materials objective into feedback for the agent. A single-objective reward may represent phase stability, formation energy, yield strength, transformation enthalpy, catalytic activity, or another target. In most HEM applications, however, several competing objectives must be considered. A generic multi-objective reward can be written as12
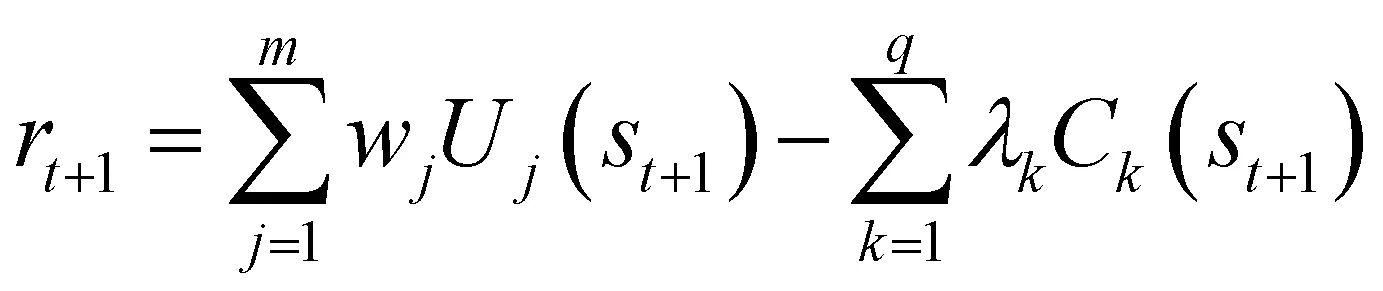
where *U*_*j*_ are normalized utility functions for desired properties, *C*_*k*_ are penalty functions for constraints or undesirable outcomes, and *w*_*j*_ and *λ*_*k*_ are weighting coefficients. Normalization is important because strength, porosity, activity, cost, and other quantities generally have different units and numerical scales. Penalty terms may be used for thermodynamic instability, poor synthesizability, excessive cost, unsafe processing conditions, or predictions outside the applicability domain of a surrogate model.

#### 4.6.2 Illustrative closed-loop HEM processing with example

A useful HEM-specific illustration is sequential process optimization during additive manufacturing. Consider laser powder bed fusion of a high-entropy alloy in which the objective is to improve mechanical performance while suppressing processing defects. The RL formulation and its interpretation are presented in Table 4. For example, if a current processing condition produces acceptable density but inadequate mechanical performance, the agent can select a permissible modification to a process parameter, observe the resulting microstructure and properties, calculate the reward, and use that transition to update subsequent decisions. This is an illustrative formulation, not evidence that any particular numerical processing window or reward weighting has already been validated experimentally with RL. In real implementation, allowable parameter ranges, measurement uncertainty, safety constraints, and reward weights must be calibrated for the specific alloy and manufacturing platform.

**Table 4:** Different components and their interpretation in RL

MDP component	Illustrative HEM interpretation
State s_*t*_	Current composition, laser power, scan speed, layer thickness, thermal history, phase information, porosity, grain size, measured properties, and predictive uncertainty
Action a_*t*_	Physically allowed adjustment of laser power, scan speed, layer thickness, heat-treatment conditions, or another processing variable
Transition *P*	Response obtained from the next fabrication/characterization step or predicted by a validated process surrogate
Reward *r*_*t*+1_	Normalized utility combining target mechanical properties and defect suppression, with penalties for cost, excessive energy use, unsafe conditions, or constraint violation
Policy π(a∣s)	Decision rule that selects the next processing modification from the current material/process state
Environment	LPBF experiment, validated AM simulator/surrogate, or automated synthesis–characterization loop

The same MDP concept can be applied to compositional design. In this case, the state contains the current alloy composition and available property information, the action changes one or more composition variables within physical constraints, and the reward is calculated from measured or predicted performance. Xian *et al.* demonstrated a closed-loop RL strategy for multicomponent alloy composition design, integrating the agent with manual synthesis and characterization and showing that RL can be coupled directly to experimental feedback in a materials-design problem.^61^

The application of RL to HEMs is no longer purely conceptual, although the literature remains much smaller than that for supervised learning or Bayesian optimization. Xian *et al.* subsequently evaluated model-based and on-the-fly RL using real-world high-entropy-alloy data and showed that the framework can operate in increasingly high-dimensional composition spaces. Their model-based formulation allows the agent to interact with a surrogate representation of the materials landscape, whereas the on-the-fly formulation can use direct experimental measurements as reward feedback.^61^ This distinction is important because it shows that RL does not require every learning step to be a physical experiment; surrogate environments can be used to reduce the experimental burden, although their uncertainty and applicability domain must be monitored.

At the nanoscale, Mubeen and Chen applied deep RL to Ag–Pd-based multimetallic nanoclusters, including high-entropy alloy systems, to accelerate the search for low-energy configurations.^62^ Farooq and Chen further demonstrated a deep-RL framework for quaternary and quinary high-entropy nanoclusters in which the agent explored complex potential-energy landscapes through stochastic structure generation and atomic displacements; candidate structures were subsequently assessed using first-principles calculations and molecular dynamics simulations.^63^ These studies provide direct examples of physically meaningful state-action-reward formulations in high-entropy systems rather than relying on generic navigation or game-based analogies.

## 5 Data quality, validation and uncertainty in AL/ML integration with HEM research

The reliability and generalizability of AI/ML models for HEMs depend not only on algorithm selection but also critically on the quality, diversity, independence, and provenance of the underlying data.^40,64^ HEM datasets are commonly assembled from heterogeneous sources, including experimental literature, high-throughput calculations, thermodynamic databases, DFT, and materials repositories. Consequently, apparently similar records may correspond to substantially different synthesis routes, heat-treatment conditions, testing temperatures, strain rates, particle sizes, phase-identification methods, or microstructural states.^18^ Before model development, the provenance of each observation should therefore be traceable, preferably including its original source, nominal and measured composition, processing history, characterization conditions, and uncertainty associated with the reported properties or phase label. Data curation should include unit harmonization, treatment of missing values, identification of inconsistent or physically implausible observations, standardization of material nomenclature, and detection of duplicate or near-duplicate records.^29^ Various AI/ML techniques have been developed and applied to the processing of HEMs to enable accurate predictions and effective optimization.

### 5.1 Dataset duplication, missing metadata, and class imbalance

Duplicate removal requires particular care in HEM datasets because identical nominal compositions do not necessarily constitute redundant observations. A given alloy, oxide, carbide, or nitride synthesized using different annealing temperatures, cooling rates, pressures, deposition conditions, or processing routes may exhibit distinct phase assemblages, microstructures, and functional properties.^18,65^ Such observations should therefore be retained when their processing and characterization conditions differ and are adequately documented. In contrast, exact duplicates arising from repeated database entries, secondary compilations, or multiple reports of the same experimental measurement should be identified to avoid artificial inflation of dataset size and statistical over-representation.

Missing processing and microstructural information are equally important. Composition-only datasets may implicitly treat materials synthesized under different conditions as equivalent, even when their grain size, phase fraction, precipitation state, porosity, defect structure, short-range order, residual stress, or segregation behavior differs substantially. Missing metadata should therefore be explicitly identified rather than silently interpreted as a common processing state. When such variables are available, they should be incorporated into model inputs to better capture the complete composition–processing–structure–property relationship.^18,66^

For classification problems, such as distinguishing single-phase solid solutions from multiphase or amorphous materials, class imbalance should also be quantified before training. A model can achieve apparently high overall accuracy by preferentially predicting an over-represented class while performing poorly for less frequent but scientifically important material states. Performance should therefore be reported using class-sensitive metrics such as precision, recall, F1-score, balanced accuracy, Matthews correlation coefficient, and precision–recall characteristics, in addition to conventional accuracy.^67^ Stratified sampling and, where appropriate, class-weighted learning may also be used to reduce bias toward dominant classes.

### 5.2 Validation beyond random train–test splitting

The strategy used to partition data is one of the most important determinants of whether reported ML performance reflects genuine materials-discovery capability. Conventional random train–test splitting assumes that training and test observations are sufficiently independent. However, HEM datasets often contain compositionally related families in which materials differ only slightly in elemental fractions. Random splitting may therefore place chemically near-identical compositions in both the training and test sets. Under such conditions, the model is primarily evaluated on interpolation among familiar compositions rather than on its ability to predict genuinely unexplored chemical systems. This form of chemical or compositional leakage can yield an overly optimistic estimate of model generalization.^64^

For HEM discovery, chemically grouped cross-validation offers a more rigorous alternative. Compositions belonging to the same elemental family or chemically related system are assigned to the same fold, preventing closely related members from occurring simultaneously in the training and test subsets. A still more stringent strategy is leave-one-system-out (LOSO) validation, in which an entire compositional or elemental family is excluded during model development and subsequently used as an independent test system. Such validation more directly assesses whether the trained model can transfer learned relationships to an unfamiliar region of chemical space.

This distinction between interpolation and extrapolation is particularly important in AI-guided materials discovery. Transferable ML interatomic potentials can accelerate atomistic exploration, but chemical-space coverage and out-of-domain validation remain central limitations. Wen *et al.* developed a general neural-network potential for C/H/N/O energetic materials using transfer learning and DFT reference data.^68^ Because “HEM” in that study refers to high-energy materials rather than high-entropy materials, it should be cited only as a methodological analogue illustrating transferable-potential construction, training-set coverage and validation, not as direct evidence for high-entropy systems. Interpolation evaluates predictions within a compositional or processing region represented in the training data, whereas extrapolation concerns predictions for material families, chemical combinations, or processing regimes absent from model construction. Because the ultimate objective of HEM informatics is often to identify candidate materials outside the experimentally explored database, extrapolative performance can be more informative than high accuracy obtained from purely random splitting.^64^ A useful hierarchy of validation strategies for HEM models is given in Table 5: The validation protocol should therefore be selected according to the intended use of the model rather than solely on the basis of which procedure provides the highest performance metric.

**Table 5:** Validation strategies used in different AI/ML techniques

Validation strategy	Principle	Major consideration for HEMs	Recommended role
Random train–test split	Samples are assigned randomly to training and test sets	Compositionally similar materials may occur in both subsets, leading to chemical leakage	Preliminary model development or interpolation benchmarking
Random *k*-fold cross-validation	Data are repeatedly partitioned into random folds	Improves statistical robustness but may still retain chemically related compositions across folds	Comparison of models within an already represented chemical domain
Chemically grouped cross-validation	Related compositions or material families are kept within the same group/fold	Reduces leakage between chemically similar materials	Preferred when compositional families can be identified
Leave-one-system-out validation	An entire alloy/material system is withheld for testing	Directly evaluates transfer to a previously unseen material family	Strong test of chemical extrapolation
Cluster-based validation	Groups are generated from compositional or descriptor-space similarity	Useful when material-family boundaries are unclear	Alternative for heterogeneous or mixed HEM datasets
External/prospective validation	Model is evaluated on independent literature data, new simulations, or newly synthesized materials	Provides evidence independent of model construction and internal data partitioning	Strongest assessment of practical generalization

### 5.3 Prevention of preprocessing and model-selection leakage

Data leakage can also occur during preprocessing and hyperparameter optimization. Operations such as missing-value imputation, normalization or standardization, feature selection, dimensionality reduction, descriptor screening, and resampling should be fitted exclusively on the training data within each validation fold and subsequently applied to the held-out samples. Performing these operations on the complete dataset before splitting allows information from the test set to influence model construction and can lead to artificially optimistic performance estimates.

Similarly, repeated hyperparameter tuning on the same test data compromises the independence of model evaluation. When extensive model selection is required, nested cross-validation provides a more rigorous strategy in which the inner loop is used for hyperparameter optimization and the outer loop provides an independent estimate of generalization performance.^64^ Such practices are particularly important for HEM datasets, which are frequently small relative to the dimensionality of the chemical and processing design space.

### 5.4 Experimental-label uncertainty and uncertainty quantification

Prediction accuracy alone is insufficient when AI models are used to guide costly HEM synthesis, DFT calculations, characterization, or processing experiments. Experimental property values and phase labels themselves may contain uncertainty arising from measurement variability, synthesis fluctuations, sample heterogeneity, characterization resolution, and differences in processing history. ML predictions should therefore, where feasible, be accompanied by estimates of predictive uncertainty.

Two conceptually distinct uncertainty sources are particularly relevant. Aleatoric uncertainty reflects irreducible variability or noise in observations, including experimental measurement error, uncontrolled synthesis variations, heterogeneous microstructures, and uncertain phase assignments. Epistemic uncertainty, in contrast, reflects incomplete knowledge of the underlying composition–processing–structure–property relationship resulting from limited or non-representative training data.^69^ Epistemic uncertainty is expected to increase when the model encounters materials that are chemically or structurally distant from those used during training and, unlike aleatoric uncertainty, can potentially be reduced by acquiring additional informative data.

Depending on the model architecture and dataset size, uncertainty may be estimated using Gaussian process predictive distributions, Bayesian approaches, bootstrap or ensemble methods, Monte Carlo dropout, conformal prediction, or related probabilistic techniques. Ensemble disagreement can provide a practical indicator of model uncertainty, whereas Gaussian process models naturally provide both a predicted mean and associated predictive variance. Such uncertainty estimates are especially valuable in active-learning and Bayesian-optimization workflows, where they can guide the selection of new experiments or simulations expected to provide maximum information gain.

### 5.5 Domain of applicability and out-of-distribution prediction

Uncertainty assessment should be interpreted together with the model's domain of applicability (DoA), namely, the region of feature space in which predictions are adequately supported by the training data. For HEM models, the applicability domain should not be defined solely by elemental fractions. Depending on the target property and modelling objective, it may encompass several complementary dimensions:

• Compositional domain: elemental combinations, concentration ranges, thermodynamic descriptors, and electronic descriptors represented during training.

• Material-family domain: HEAs, HEOs, HECs, HENs, refractory systems, transition-metal-rich systems, or other families included in the training set.

• Processing domain: synthesis route, annealing history, cooling rate, pressure, additive-manufacturing parameters, deposition conditions, or other processing variables.

• Microstructural domain: grain size, phase fraction, porosity, precipitation state, short-range order, defect density, segregation, or related structural characteristics.

• Property domain: numerical range and physical conditions over which the target property has been measured or calculated.

• Nanostructural/surface domain: particle size, morphology, agglomeration, oxidation state, surface segregation, and support interaction where nanostructured HEMs are considered. Beitollahi *et al.* showed in CNT-reinforced nanocomposites that aggregation and length-scale assumptions can materially affect predicted structural response.^70^ Although this is not a HEM study and should therefore be treated only as a methodological analogue, it reinforces the broader modelling principle that heterogeneous particle distributions cannot always be represented by average composition or size alone.

This consideration is particularly important for generative and inverse-design models. A generated composition may satisfy the mathematical optimization objective while still occupying a region poorly supported by existing experimental or computational data. Therefore, candidate generation should be followed by applicability-domain assessment, uncertainty screening, physical and thermodynamic constraints, and synthesizability evaluation before experimental realization.

### 5.6 Independent and prospective validation

Ultimately, the most convincing demonstration of AI-model utility is validation using materials not involved in any stage of model construction or selection. Independent validation may involve external literature datasets, newly performed DFT or CALPHAD calculations, or targeted synthesis and characterization of model-recommended compositions. For AI-guided HEM discovery, prospective validation, in which the model predicts previously untested compositions before their experimental evaluation, provides substantially stronger evidence of discovery capability than retrospective agreement with randomly withheld data.

## 6 Hierarchical descriptor representation for HEM modelling

The predictive performance and physical transferability of an AI model depend not only on the learning algorithm but also on how the material is represented, as shown in Table 6. Many early HEM models relied predominantly on nominal composition and composition-averaged descriptors such as elemental fractions, VEC, atomic-size mismatch, mixing enthalpy, configurational entropy, and electronegativity differences. These descriptors remain valuable for rapid screening; however, they cannot uniquely distinguish materials with identical nominal compositions but different synthesis histories, phase fractions, grain structures, defect populations, interfaces, or nanoscale surface states. Nominal composition alone is insufficient when processing alters the microstructure that ultimately controls measured properties. Ma *et al.* showed that selective laser melted FeCoCrMnNi-based high-entropy-alloy composites undergo substantial precipitation and microstructural evolution during ageing, with the resulting mechanical response reflecting precipitation strengthening, dislocation strengthening and defect-related lattice effects.^18^ This directly supports inclusion of synthesis route, additive-manufacturing parameters, thermal history and heat treatment alongside composition in predictive HEM models. Recent studies on additively manufactured HEAs, chemical short-range order, grain-boundary segregation, and high-entropy nanoparticles demonstrate that these non-compositional variables can substantially alter phase stability and functional response.^30,71–75^

**Table 6:** Hierarchical descriptor framework for AI/ML modelling and inverse design of HEMs

Descriptor level	Representative inputs	Why it matters/typical use
Composition	Elemental fractions (*x*_*i*_), VEC, atomic-size mismatch (*δ*), Δ*H*_mix_, Δ*S*_mix_, electronegativity/electronic differences	Rapid screening of chemistry and empirical phase/property trends; provides baseline representation but not processing or structural history
Processing	Synthesis route, temperature, time, cooling rate, pressure, atmosphere, heat treatment, deformation history, AM laser power/scan speed/hatch spacing/energy density	Distinguishes materials with the same nominal composition but different thermal or manufacturing histories; critical for process–structure–property modelling^73,75^
Microstructure	Grain size, phase identity and phase fraction, chemical short-range order (SRO), precipitation state, texture, porosity, dislocation/defect density	Captures experimentally realized structure and local ordering that directly influence strength, ductility, transport and phase stability^74^
Interface	Grain-boundary chemistry, segregation/co-segregation, boundary character, interfacial energy, local strain and residual stress	Represents interfacial phenomena that can control cohesion, diffusion, precipitation, corrosion and microstructural evolution^72^
Nanostructure	Particle size, morphology/shape, facet exposure, aspect ratio, agglomeration state, surface area, pore architecture	Essential for high-entropy nanoparticles, where size and morphology alter stability, active-site distribution, transport and catalytic response
Surface chemistry	Oxidation state, surface composition/segregation, adsorbate coverage, support interaction, interfacial charge transfer, d-band/electronic surface descriptors	Critical for catalysis and electrochemistry because activity is governed by the chemically active surface rather than bulk composition alone^71^

A more physically complete representation can therefore be organized as a hierarchy of application-relevant descriptor groups spanning composition, processing, microstructure, interface, nanostructure, surface chemistry, and physics-based information. This hierarchy is conceptual rather than prescriptive: not every model requires all seven levels, and the descriptor subset should be selected according to the target properties, data availability, intended domain of application, and risk of overfitting.

This hierarchy also provides a practical basis for deciding when composition-only ML is adequate and when higher-level descriptors are necessary. For example, a model intended only for preliminary screening of equilibrium phase tendencies may rely mainly on composition and thermodynamic descriptors. In contrast, a model predicting the mechanical response of an additively manufactured HEA should incorporate process variables and microstructural descriptors because laser power, scan speed, cooling conditions, porosity, phase fraction, grain size, and residual stress can all influence the measured response.^73,75^ Interfacial and microstructural descriptors are particularly important when segregation or precipitation changes deformation mechanisms. Luo *et al.* demonstrated in NbMoTaW-based refractory HEAs that interstitial carbon promotes chemical short-range order, grain-boundary enrichment and, at higher carbon contents, carbide formation, producing distinct strength–ductility responses.^65^ This provides direct evidence that grain-boundary chemistry, segregation state, CSRO and second-phase evolution should be represented where models seek to predict or optimize mechanical performance. Likewise, modelling catalytic high-entropy nanoparticles requires descriptors of particle size, shape, surface segregation, oxidation state, and support interaction because these determine the population and electronic structure of active surface sites.^30,71^

The hierarchy should not be interpreted as requiring indiscriminate inclusion of every available feature. Excessive or highly correlated descriptors can increase model variance, reduce interpretability, and aggravate overfitting in small HEM datasets. Descriptor selection should therefore combine materials-domain knowledge with feature selection, dimensionality reduction, regularization, and leakage-resistant validation. Where descriptors are missing systematically, for example, unreported heat-treatment histories or unavailable nanoparticle surface states, the resulting limitation should be treated as a domain-of-applicability issue rather than implicitly assuming that nominal composition contains the missing information.

For inverse design, the same framework provides an additional advantage: descriptors can be separated into variables that are directly controllable during synthesis (composition and processing), variables that emerge from those decisions (microstructure, interface and surface state), and physics-based latent or calculated quantities that constrain feasible solutions. This distinction enables AI-guided design to move from simple composition optimization toward experimentally actionable composition–processing–structure design.

## 7 Case study of HEMs with AI/ML

AI/ML models are commonly employed to predict phase formation, estimate mechanical properties, identify novel compositions, and guide experiments through active learning strategies. In the context of HEMs, these models are broadly applied across the following categories, particularly with a view toward their future research and technological applications.

### 7.1 Phase prediction in HEMs

Phase prediction plays a very crucial role in HEMs because the mechanical and functional performance of these materials is directly related to their phase stability. It is necessary to identify whether a multicomponent composition is a single solid-solution composition, multiple phases or an amorphous structure. The conceptualization of phase formation in HEMs has been shifting towards data-based methodologies and first principles-based concepts, as opposed to the empirical design concepts. To formulate a powerful paradigm for predicting phase formation in HEAs, Zhang *et al.* suggested several semi-empirical thermodynamic measures, including atomic size mismatch (*δ*), mixing enthalpy (Δ*H*_mix_), mixing entropy (Δ*S*_mix_), the *Ω* parameter, and VEC.^10^ Miracle *et al.* proposed the structural relations, or cluster-type models, which offered a more generalized view of the formation of phases and the position of atoms in the complex alloys. Since these models are primarily based on experimental data, they are limited in their ability to predict phase formation in non-equiatomic systems or by pure empiricism.^8^ The work explains the atomic packing, short-range ordering and local chemical interactions, which oppose the previous belief that the configurational entropy is the sole determining factor of solid solution stabilization in random alloys. Although this work represents a step towards more empirical rules, it is to a great extent qualitative and does not provide a strong predictive scheme. Based on this, Oliynyk *et al.* continued the work by developing data-driven phase prediction through large experimental data. They used the regression models that included the elemental descriptors such as electronegativity difference, atomic radius mismatch, and VEC to predict the phase stability with a reasonable degree of accuracy.^76^ Their methodology is more quantitative and systematic and predicts phases by the establishment of statistical correlations, despite the fact that more sophisticated mechanistic pathways have been established. This methodology is limited by the fact that only limited experimental data are available. To overcome this problem, Wen *et al.* enlarged the prediction of the phase using ML algorithms. Their approach makes use of curated experimental data combined with a wide range of thermodynamic and elemental descriptors to boost the predictive power of phase formation in complex alloy systems.^77^ The research shows enhanced predictive precision and, in the process, they are able to capture the complicated nonlinear interactions between governing variables. It also points out the effectiveness of materials informatics as a strong method of the prompt screening of broad compositional areas. Kaufmann *et al.* more recently embraced physics based computational techniques incorporating DFT into thermodynamic modeling frameworks. Despite the lack of a large body of experimental data, this method can be used to make first-principles-based predictions of phase stability, as well as determine potentially unstable or metastable phases, as illustrated in Fig. 7a.^78^ It is a highly predictive method that is based on the principles of fundamental physics and is especially useful when exploring unexplored compositional space; however, it requires a large amount of computing power. Most importantly, the above methods are not mutually exclusive but complementary. Empirical rules help screen quickly at the initial stage, structural models give mechanistic insight, data-driven approaches allow large-scale compositional discovery, and first-principles computations can be used to give high accuracy validation. Taken together, these strategies provide a solid base for an integrated framework of phase formation prediction in HEMs.

**Fig. 7:**
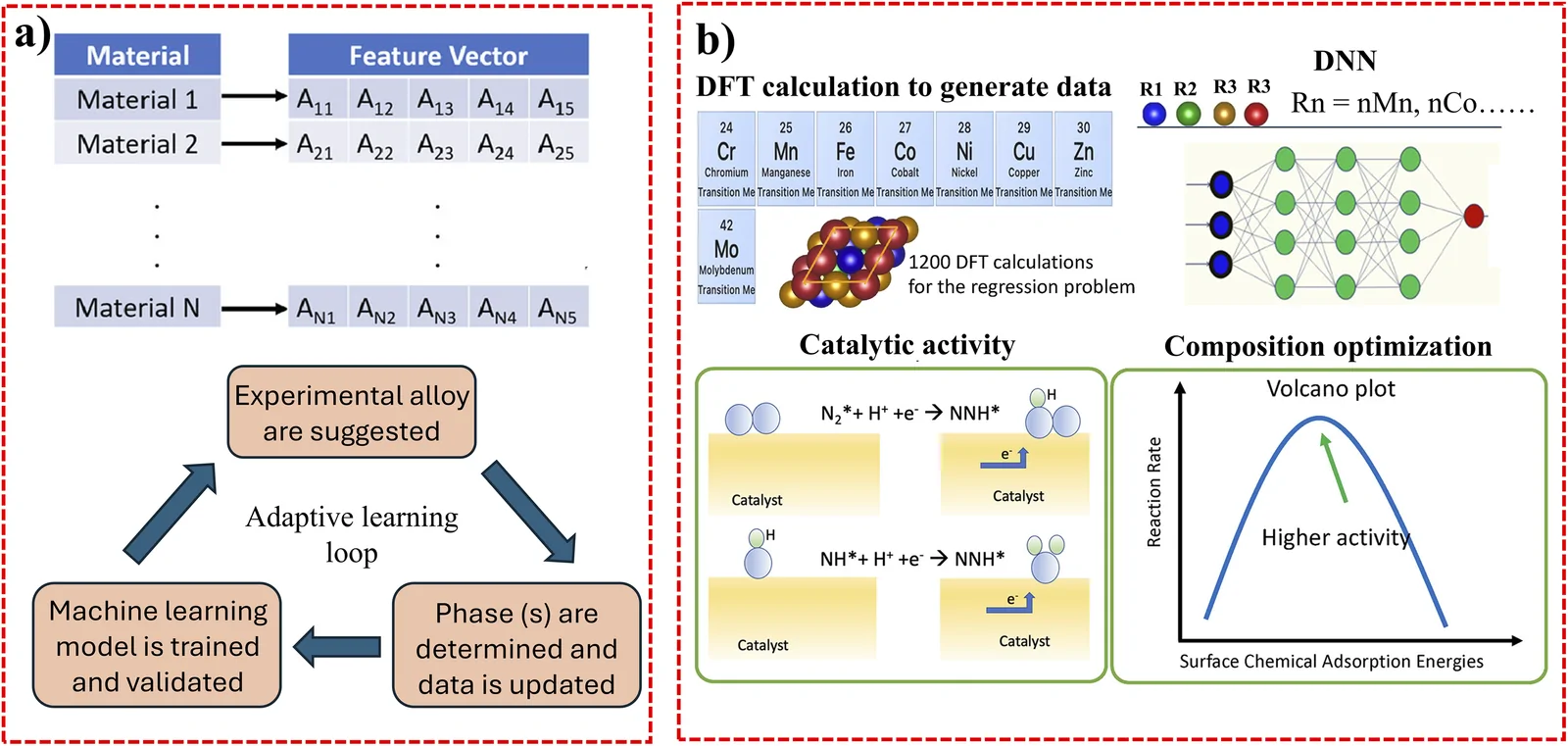
Use of AI/ML models for (a) phase prediction, adapted from ref. 78 with permission from Elsevier, copyright 2020. (b) Schematic illustration of the steps used to optimize HEA catalysts for the nitrogen reduction reaction in a water medium, adapted from ref. 79 with permission from American Chemical Society, copyright 2024.

### 7.2 Mechanical property prediction in HEMs

Mechanical property prediction in HEMs has become one of the most effective applications of ML, in part because of the relative accessibility and effectiveness of data-driven modeling methods. Historically, however, CALPHAD simulations and experimental research have been the primary reliable tools for alloy property evaluation.^8^ However, the vast expanse of composition that the presence of five or more principal elements creates renders exhaustive exploration of experiments impractical. With the use of ML methods, composition, processing conditions, and microstructural characteristics can be used as key inputs to predict mechanical properties such as yield strength, hardness, and ductility. Gradient boosting, random forest and neural networks are examples of supervised learning models that have been trained using experimental data to achieve high predictive accuracy. Wen *et al.* developed an ML-surrogate/experimental-design workflow for hardness optimization in an Al–Co–Cr–Cu–Fe–Ni HEA system. The primary-source abstract reports that several alloys with hardness approximately 10% higher than the best value in the original training set were obtained through seven experiments.^77^ Machine-learning models have shown promising performance for predicting mechanical properties of high-entropy alloys, but reported accuracy is strongly dependent on the target properties, dataset, descriptors, and validation protocol. For example, Lee *et al.* trained composition-based models on 181 HEA yield-strength data points; using an 80:20 train–test split with hyperparameter tuning, gradient boosting achieved a test-set *R*^2^ = 0.8538 (RMSE ≈ 192.99 MPa), with additional validation against external HEA categories.^80^ Such values should be interpreted within the chemical and processing domain represented by the training data rather than as universal HEA prediction accuracy.

In HEMs, multiple mechanical phenomena, including the quality of solid-solution, degree of lattice distortion, and short-range ordering effects influence overall performance. These mechanisms are associated with factors such as atomic radius mismatch, valence electron concentration, mixing enthalpy, and electronic structure characteristics. ML methods effectively integrate these interrelated descriptors, enabling the resolution of challenging property prediction problems.^81^

Crystal Graph Convolutional Neural Networks (CGCNNs), pioneered by Xie and Grossman, encode the crystal structures by considering both atomic properties and the relative distances between neighbors. This allows the model to capture the lattice distortion effect inherently, rather than relying on an average parameter.^82^ Recently, hybrid approaches coupling mechanistic models with Bayesian or ensemble ML have further helped in identifying the uncertainty quantification as well as the effect of physical parameters.

### 7.3 Thermal property prediction

For high-temperature structural applications, thermal and mechanical properties are strongly interdependent; therefore, their simultaneous optimization using ML is critically important. One study demonstrated that ML-based models can predict the bulk modulus of HEAs more accurately than traditional approaches, facilitating the design of alloys with enhanced bulk modulus values. Overall, these investigations indicate that data-driven methodologies are highly versatile across a broad range of thermophysical properties, including the prediction of thermal stability and hydrogen storage performance.^83^ ML models have enabled the identification of complex nonlinear correlations among parameters such as configurational entropy, atomic size mismatch, and electronic structure complexity, all of which significantly influence thermal stability. VAEs have been combined with Bayesian optimization methods to find compositions with significantly lower thermal expansion coefficients in refractory HEAs. The model can also produce interpolated alloy compositions that can fill the gaps between existing well-known Cantor-type systems by traveling through the latent space. Moreover, other wide-ranging descriptors such as atomic mass, melting temperature, Debye temperature, and differences between electronegativity may be added to ML models to increase further predictive ability. Interestingly, ML models can be used to predict a variety of thermomechanical properties at once, as the models are capable of identifying optimal materials that trade off density, strength, and thermal stability.^81^ Radiation tolerance in compositionally complex alloys is strongly dependent on alloy chemistry and irradiation conditions.

### 7.4 Extreme-environment and radiation-tolerance prediction

HEMs are being investigated for nuclear and other extreme-environment applications because chemical complexity can modify defect transport, damage accumulation, phase stability, and irradiation-induced microstructural evolution. Experimental and modelling studies on concentrated solid-solution alloys have shown that increasing chemical complexity can suppress the accumulation and mobility of irradiation-induced defects relative to simpler elemental systems. For example, Granberg *et al.* reported substantially reduced irradiation-damage accumulation in single-phase NiFe and NiCoCr alloys compared with elemental Ni, without observable irradiation-induced phase separation or amorphization under the studied conditions.^84^ These results support the potential of compositionally complex alloys for radiation-tolerant applications, although the magnitude of the benefit remains dependent on alloy chemistry, irradiation dose, temperature, particle species, and microstructural state.

Radiation response is intrinsically multiscale: energetic displacement events generate point defects and defect clusters, which subsequently migrate, interact, and evolve over time. Osetsky *et al.* used microsecond-scale molecular dynamics together with kinetic Monte Carlo and mean-field modelling to show that chemical complexity can produce chemically biased and sluggish interstitial diffusion in concentrated Ni–Fe alloys.^85^ Such results illustrate why local chemical environments and defect energetics are important descriptors for modelling irradiation response in multicomponent alloys. Lu *et al.* reported markedly reduced void swelling in equiatomic concentrated solid-solution alloys under Ni-ion irradiation at 773 K; under representative conditions, swelling decreased from approximately 1.8% in Ni to approximately 0.02% in NiFe/NiCoFeCr, and from approximately 9.4% in Ni to approximately 0.1% in NiCoFeCrMn at a higher irradiation dose.^86^ These results demonstrate up to roughly two orders of magnitude improvement under the reported conditions, rather than a universal swelling-reduction factor for all HEMs. Radiation damage is computationally costly, and it may need large-scale Molecular Dynamics (MD). Training ML Interatomic Potentials (MLIPs) on DFT data allows researchers to simulate the evolution of displacement cascades over longer timescales and larger volumes than was previously possible.^85^

ML can complement these approaches by linking irradiation conditions, composition and microstructural descriptors to measured response, by constructing high-dimensional interatomic potentials from first-principles reference data, and by accelerating models of defect migration and evolution. Zhao summarized these directions as ML-based irradiation-response prediction, ML-based interatomic-potential development, and ML-informed defect-evolution modelling.^87^ However, direct ML prediction of irradiation response in high-entropy materials remains considerably less mature than phase or mechanical-property prediction, largely because irradiation datasets are sparse and highly condition-dependent. Consequently, radiation-focused models should explicitly retain dose, dose rate, irradiation temperature, particle type/energy, initial microstructure, defect state and uncertainty rather than relying on nominal composition alone.

ML interatomic potentials are particularly relevant to this problem because they can approach first-principles accuracy while enabling atomistic simulations at length and timescales that are difficult to access routinely with DFT. Recent HEA-specific MLIP developments demonstrate the feasibility of constructing potentials for chemically complex alloy spaces.^84^ For radiation-damage applications, however, their transferability must be tested carefully for high-energy collision configurations, defects, interfaces and temperatures outside the training distribution.

### 7.5 Functional property prediction

HEMs are also used extensively in catalysis, nuclear and energy-related applications due to their better corrosion resistance, catalytic activity, and radiation tolerance. The determination of these properties is, however, complex in nature due to the complicated and at times aberrant interactions that take place during electrochemical and surface processes. The ML approaches address this problem by integrating experimental data with the data obtained through simulation to model the underlying behavior. For example, ML models trained on experimental data have been used to successfully predict corrosion rate and current density. Data-driven models have been used to predict corrosion descriptors of multi-principal element alloys, although reported accuracy depends strongly on the target and dataset construction. In a study based on 619 experimental MPEA corrosion entries, Ghorbani *et al.* reported a random forest test score of approximately *R*^2^ = 0.66 for corrosion potential (*E*_corr_) using the experimental dataset; after augmentation, the reported test score increased to approximately *R*^2^ = 0.90. The same study obtained different performance levels for corrosion current density (*i*_corr_) and pitting potential (*E*_pit_), emphasizing that a single *R*^2^ value should not be generalized across corrosion properties.^88^ Other than corrosion protection, HEMs have also been investigated in the field of catalysis. Quantum mechanical data have been used to screen potential electrocatalysts to reactions including ammonia synthesis using neural networks, allowing the discovery of a highly efficient, multi-complex multi-element surface (Fig. 7b).^79^ The progress of the sustainable energy technologies is the creation of cost-efficient and long-lasting electrocatalysts to evolve the Hydrogen Evolution Reaction (HER) and Oxygen Evolution Reaction (OER). The traditional catalysts normally rely on noble metals that are not abundant such as Pt or RuO_2_. In comparison, HEM catalysts, especially oxides and high entropy alloys, take advantage of the cocktail effect to produce a wide range of adsorption sites of varying binding energies. High-entropy alloy surfaces provide a broad distribution of local adsorption environments that can be exploited for catalyst screening. Batchelor *et al.* used first-principles adsorption-energy modelling to demonstrate the potential of multicomponent alloy surfaces as a discovery platform for the oxygen reduction reaction, illustrating how the diversity of local surface sites can be searched computationally for favorable catalytic environments.^89^

## 8 Present capability and future HEM-AI systems

AI-driven HEM research is progressing through distinct levels of maturity, as shown in Table 7. Supervised phase/property prediction, active-learning-guided screening, and hybrid ML-DFT/CALPHAD workflows are already demonstrated in high-entropy alloys. Generative inverse design, machine-learned interatomic potentials, multi-objective Bayesian optimization, and closed-loop design–make–test–learn workflows emerge rapidly but still require broader validation across composition and processing domains. Fully autonomous HEM laboratories, LLM-mediated scientific reasoning, and comprehensive multiscale digital twins remain longer-term system-level objectives, as shown in Fig. 8. Their realization will depend on reliable synthesizability constraints, uncertainty quantification, applicability-domain control, automated characterization, external validation, scale-up, and robust experimental closure. Accordingly, AI should be viewed as progressively augmenting and automating HEM discovery rather than having already eliminated the scientific and engineering bottlenecks associated with materials development.

**Table 7:** Maturity framework for AI-enabled design and discovery of HEMs

Maturity level	Representative capabilities	Evidence and appropriate interpretation	Key requirements before broader adoption
Demonstrated	Supervised ML for phase/property prediction; active-learning-guided composition screening; ML integrated with DFT/CALPHAD and targeted experiments	Multiple HEA studies have reported phase/property models with experimental validation, and active learning has been used in a closed loop to discover high-entropy Invar alloys from a vast compositional space.^11,45,46^ These methods can be described as established research capabilities, while their transferability remains dataset- and domain-dependent	Chemically independent validation; uncertainty estimation; provenance-aware datasets; explicit processing/microstructure descriptors
Emerging	Generative inverse design; ML interatomic potentials (MLIPs); multi-objective Bayesian optimization; RL-guided search; HEM-specific design–make–test–learn loops	Direct HEM/HEA examples now exist for generative models, MLIPs and multi-objective Bayesian search, including experimental or high-fidelity computational campaigns.^52,74^ However, performance is not yet sufficiently standardized across HEM families to treat these workflows as routine	Synthesizability constraints; model calibration; applicability-domain control; multi-fidelity validation; robust experimental feedback
Prospective	Fully autonomous HEM laboratories; LLM-controlled hypothesis generation and experimental reasoning; comprehensive multiscale HEM digital twins; autonomous scale-up	Autonomous inorganic-materials synthesis and LLM-directed chemistry have been demonstrated outside HEM-specific workflows,^90,91^ and digital twins are advancing across materials engineering.^92^ Their comprehensive, reliable integration for multiscale HEM discovery and manufacturing remains prospective	Safety/governance; causal reasoning; cross-platform interoperability; real-time characterization; uncertainty propagation; reproducible scale-up and external validation

**Fig. 8:**
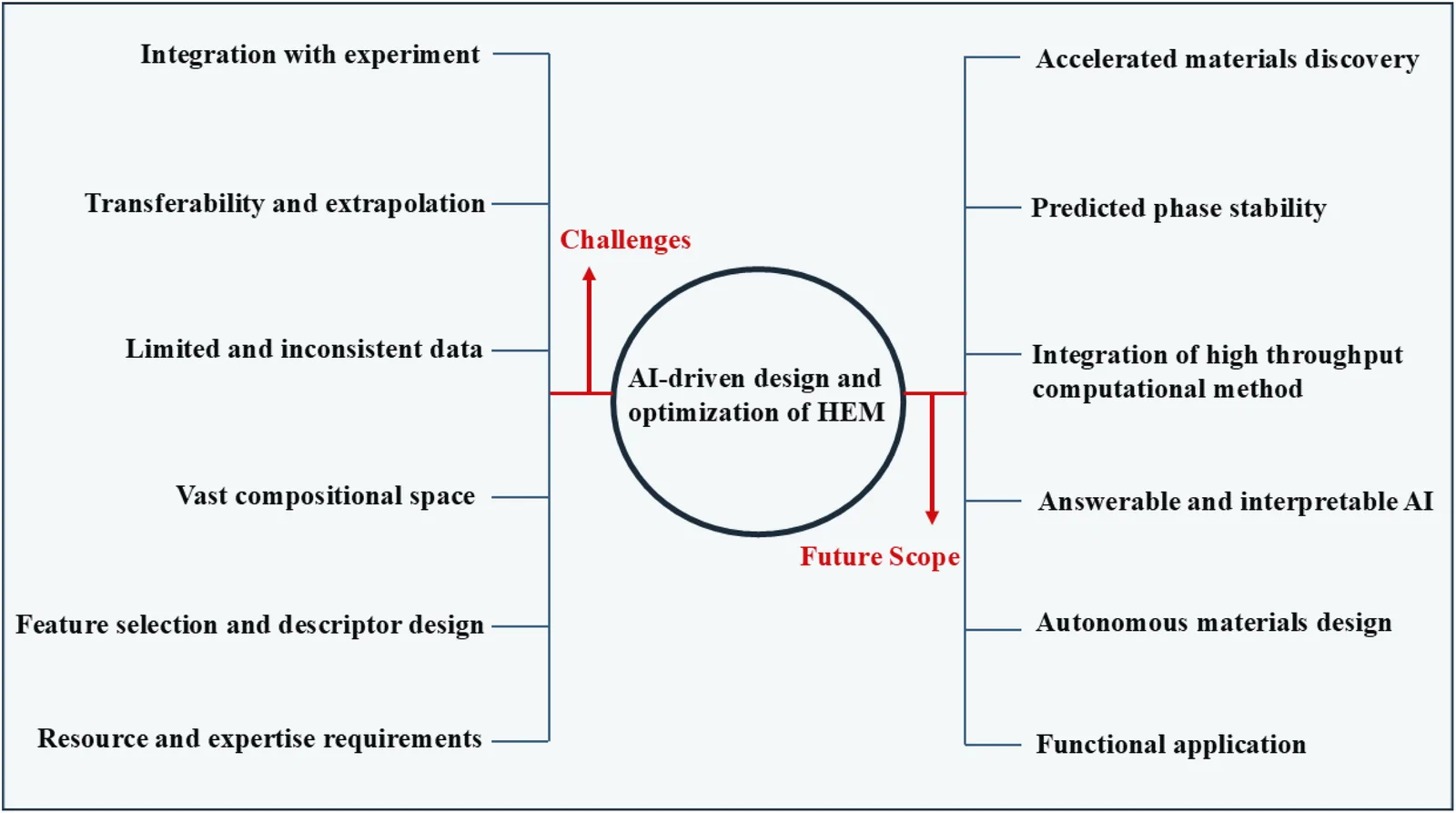
Challenges and future scope of AI-driven design and performance of HEMs.

## 9 Conclusion

High-entropy materials provide a chemically diverse platform in which composition, processing, microstructure, interfaces, and local chemical environments jointly determine phase stability and functional performance. AI and machine-learning methods have already demonstrated practical value in several parts of this design problem, including phase classification, prediction of selected mechanical and functional properties, composition screening, active-learning-guided alloy discovery, and the integration of data-driven models with density-functional theory, CALPHAD, atomistic simulations, and targeted experiments. These developments show that AI can assist the prioritization of candidate materials and reveal nonlinear relationships that are difficult to resolve using composition-based empirical rules alone.

The predictive reliability of current models, however, remains strongly dependent on the quality and scope of the available data. HEM datasets are often small, heterogeneous, and assembled from studies employing different synthesis routes, heat treatments, characterization procedures, and property-testing conditions. Models based primarily on nominal composition may also omit important variables such as phase fraction, grain size, short-range order, precipitation, porosity, grain-boundary segregation, particle morphology, oxidation state, and surface or support interactions. In addition, high accuracy obtained from random train–test splits does not necessarily establish extrapolative performance for chemically distinct material families. Uncertainty, applicability domain, and external validation should therefore be considered alongside conventional accuracy metrics.

Further progress will require improvements in both data infrastructure and model design. Provenance-aware databases, standardized reporting of processing and microstructural information, removal of duplicate or inconsistent records, and leakage-resistant validation are necessary for more reliable benchmarking. Hierarchical descriptors that connect composition with processing, microstructure, interfaces, nanostructure, surface chemistry, and physics-based quantities should improve the physical relevance of predictive and inverse models. Uncertainty quantification, out-of-distribution detection, multi-fidelity modelling, physics-informed constraints, and explicit treatment of synthesizability and processing feasibility will be particularly important when AI is used to recommend unexplored compositions.

Within these constraints, closer integration of AI with thermodynamic modelling, first-principles and atomistic calculations, high-throughput synthesis, automated characterization, and iterative experimental feedback may enable more efficient and reproducible exploration of high-entropy materials. Emerging approaches such as generative inverse design, machine-learned interatomic potentials, multi-objective Bayesian optimization, and closed-loop experimentation are promising, but their broader utility will depend on independent validation and transfer across composition, processing, and length scales. Thus, the near-term role of AI in HEM research is best viewed as an increasingly capable decision-support and design framework that complements physical modelling and experimentation, with higher levels of autonomy becoming scientifically meaningful only as data quality, model reliability, synthesizability assessment, and experimental closure improve.

## Conflicts of interest

The authors declare no conflicts of interest.

## Data Availability

No primary research results, software or code, have been included and no new data were generated or analysed as part of this minireview.
